# A lactate–mitochondrial axis framework for mild hyperthermia and checkpoint blockade in driver-negative NSCLC: testing temporal coupling

**DOI:** 10.3389/fimmu.2026.1969238

**Published:** 2026-10-22

**Authors:** Meihan Liu, Yaoshu Song, Hongpan Zhang, Yan Gui, Daiyuan Ma

**Affiliations:** 1 North Sichuan Medical College, Nanchong, China; 2 Department of Oncology, Affiliated Hospital of North Sichuan Medical College, Nanchong, China; 3 Key Laboratory of Integrated Precision Diagnosis and Treatment for Solid Tumors, Sichuan Provincial Health Commission, Nanchong, China

**Keywords:** immune checkpoint blockade, immunometabolism, lactate metabolism, mild hyperthermia, non-small cell lung cancer, T-cell exhaustion, temporal coupling, tumor hypoxia

## Abstract

Metabolic constraints, including hypoxia, lactate, and adenosine that impair CD8^+^ T-cell function and mitochondrial fitness, are increasingly recognized as contributors to resistance to immune checkpoint inhibitors (ICIs) in driver-negative (lacking an established first-line actionable driver alteration) advanced non-small cell lung cancer (NSCLC). We propose a hypothesis-driven framework asking whether transient tissue-level metabolic relief can be temporally aligned with the slower process of T-cell reinvigoration driven by immune checkpoint blockade (ICB). Its mechanistic basis is a lactate–mitochondrial axis linking extracellular metabolic suppression to mitochondrial states in tumor-infiltrating T cells, with T-cell-intrinsic mitochondrial adaptation treated as a testable extension. Locoregional mild hyperthermia (39–42 °C) is conceptualized as a transient modulator of tissue-level metabolic gradients rather than a direct immune activator. Because oxygenation, metabolite distribution, and acid–base status may change independently or in opposing directions, oxygenation is treated as a physiological readout rather than a surrogate for metabolic relief. We define a relief window as transient attenuation of hypoxic constraint accompanied by redistribution of lactate and adenosine away from T-cell-containing regions. Because such windows are short relative to ICB-driven reinvigoration, their therapeutic relevance may depend on recurrent temporal overlap, quantified through coverage and placement using provisional criteria at graded levels of evidence. The framework is proposed to apply primarily to glycolytic, hypoxic tumors retaining reinvigoration-competent progenitor exhausted T cells, with KRAS/STK11 co-mutant and KEAP1-altered NSCLC prioritized for testing as indicators of metabolic phenotype rather than predictors of benefit, subject to baseline confirmation that a reinvigoration-competent T-cell compartment is present. We specify necessary conditions, enabling requirements, and failure conditions, together with safety monitoring for repeated heating, while treating local-to-systemic immune propagation as an optional extension. A phase 0 strategy is proposed to establish whether reproducible candidate relief windows can be detected in patients, before efficacy testing and before any inference of clinical benefit.

## 1 Introduction

Lung cancer remains the leading cause of cancer-related mortality worldwide, with non-small cell lung cancer (NSCLC) accounting for approximately 80–85% of cases (1). In metastatic NSCLC without actionable driver alterations, immune checkpoint inhibitor (ICI)-containing regimens have become central components of first-line treatment (2). A considerable proportion of patients nevertheless exhibit primary non-response or develop acquired resistance, limiting the durability of therapeutic benefit (3). Early explanations for immunotherapy failure emphasized impaired antigen presentation, insufficient tumor immunogenicity, or defective immune recognition. Increasing evidence indicates that resistance is also shaped by metabolic constraints within the tumor microenvironment (TME), where hypoxia-driven glycolysis, lactate accumulation, extracellular acidosis, and adenosine signaling limit CD8^+^ T-cell function and responsiveness to immune checkpoint blockade (ICB) (4). Hypoxia can potentiate this restriction in part by increasing extracellular adenosine, an effect reported to be reversible by correcting tumor oxygenation (5, 6). ICI resistance may therefore be conceptualized as metabolically constrained immune dysfunction in addition to a failure of immune recognition.

Metabolic suppression affects T cells at both extracellular and intracellular levels. Tumor-derived metabolites and hypoxic conditions impose persistent environmental stress, while tumor-infiltrating lymphocytes undergo mitochondrial dysfunction, impaired metabolic flexibility, and exhaustion-associated adaptation (4). We therefore propose the lactate–mitochondrial axis as a conceptual framework linking extracellular metabolic pressure to mitochondrial states within tumor-infiltrating T cells. Because progenitor exhausted CD8^+^ T cells (Tpex) retain greater metabolic competence and responsiveness to checkpoint blockade, this raises the hypothesis that transient reduction of metabolic constraints could create conditions more permissive for checkpoint-driven reinvigoration (7).

Mild hyperthermia is one potential means of modulating these constraints at the tissue level. Unlike cytotoxic thermal approaches, locoregional mild hyperthermia (39–42 °C) primarily induces physiological changes, including increased perfusion, altered oxygen delivery, and changes in vascular–interstitial exchange (8). The question addressed here is whether such transient physiological modulation can generate measurable changes in the metabolic environment experienced by immune cells. We examine this framework in driver-negative NSCLC, here denoting tumors without an established first-line actionable driver alteration, where systemic therapy relies heavily on ICI-based regimens, targeted alternatives are limited, and tumors frequently exhibit perfusion heterogeneity and hypoxia (3, 9). KRAS/STK11 co-mutant and KEAP1-altered tumors are proposed as priority subgroups for testing on the basis of their metabolic phenotype, rather than as predictors of benefit (9).

Previous reviews of hyperthermia combined with immunotherapy have, to our knowledge, largely emphasized immune activation, heat-shock responses, immunogenic cell death, and emerging clinical observations (10). A recent comprehensive example is the review by Liu et al. (10), which surveys hyperthermia modalities combined with checkpoint inhibitors across malignancies and summarizes reported clinical outcomes. The present framework differs in both scope and question. It is restricted to locoregional mild hyperthermia in driver-negative NSCLC and asks whether transient tissue-level metabolic modulation can be temporally aligned with the slower process of ICB-driven T-cell reinvigoration.

Perfusion and oxygenation changes induced by mild hyperthermia are short-lived relative to the days-to-weeks course of T-cell activation, expansion, and differentiation after checkpoint blockade (8, 11). The relevance of mild hyperthermia may therefore depend less on the magnitude of an individual thermal response than on whether repeated exposures generate relief windows that overlap with checkpoint-responsive T-cell remodeling. Whether such windows arise reproducibly in immune-relevant regions of human tumors is the central unverified premise of the framework. Improved perfusion and oxygenation are not a directionally safe assumption either, since the same changes may also support tumor-cell metabolism and growth, a possibility treated here as a failure condition rather than a caveat (Section 4.6). The contribution intended here is therefore methodological. Within this heat–metabolism–immunity framework, we formulate temporal coupling as a falsifiable hypothesis, operationalize relief windows through measurable criteria and schedule-level coverage and placement, and propose a phase 0 design to determine whether the central premise holds in patients.

## 2 Extrinsic metabolic barriers to immunotherapy efficacy

Aberrant tumor vasculature is an important upstream contributor to metabolic immunosuppression in NSCLC. Rapid tumor growth is frequently accompanied by vascular disorganization, heterogeneous perfusion, and inadequate pericyte coverage, resulting in persistent intratumoral hypoxia (12, 13). Through hypoxia-inducible factor-1α (HIF-1α)-dependent transcriptional programs, hypoxia promotes glycolytic adaptation and immunosuppressive signaling (4, 13), and tumor cells commonly exhibit enhanced aerobic glycolysis (the Warburg effect), generating lactate that is exported through monocarboxylate transporters (MCTs) and contributes to extracellular acidification (4).

Lactate also functions as a signaling metabolite. At elevated concentrations it can suppress CD8^+^ T-cell proliferation and cytokine production through intracellular acidification and disruption of metabolic enzyme activity (14), although its effects are context dependent, and it has also been reported to promote stem-like properties of CD8^+^ T cells under specific experimental conditions (15). In advanced solid tumors, where elevated lactate coexists with extracellular acidosis, glycolytic activity, and hypoxia, the net effect within immune-restricted regions appears predominantly suppressive (4, 14).

Lactate-mediated immunosuppression operates together with adenosine signaling. Under hypoxic conditions the CD39/CD73 ectonucleotidase axis is upregulated, promoting sequential conversion of extracellular ATP into adenosine (16), and adenosine signaling through the A2A receptor activates cAMP–PKA pathways, suppresses T-cell receptor (TCR) signaling, and reduces T-cell metabolic activity (16). In murine tumor models, systemic oxygen supplementation reduced hypoxia- and adenosine-associated immunosuppression and enhanced antitumor immune responses (6, 17). These findings support a functional relationship between oxygen availability and adenosine-mediated suppression, but they involve systemic oxygen administration in mouse tumors and do not establish that transient, local oxygenation changes induced by mild hyperthermia produce equivalent effects.

Quantitative modeling has examined the same interaction. A spatiotemporal model combining an agent-based framework, oxygen diffusion, and ordinary differential equations, calibrated on murine breast cancer data from dendritic-cell vaccination with adenosine or HIF-1α pathway modulation, identified adenosine-related parameters, particularly the baseline rate of hypoxia-independent adenosine production, as influential determinants of tumor growth, and predicted greater effects from combined inhibition of hypoxia and adenosine pathways than from either alone (5). The model assumed the hypoxia–adenosine relationship, was calibrated in a single tumor context, lacked direct validation of the combined condition, and incorporated neither checkpoint blockade nor hyperthermia, so it is hypothesis-generating support for the concept of coupled metabolic constraints rather than validation of the present framework.

Because these constraints are distributed across several nodes and generate spatially heterogeneous, dynamically changing gradients (18), targeting a single pathway may be insufficient, and a tissue-level intervention that modifies perfusion, oxygen delivery, and metabolite distribution may be complementary. Together with the T-cell mitochondrial adaptation described below, these extracellular constraints constitute the external component of the lactate–mitochondrial axis.

## 3 Impaired metabolic adaptation and mitochondrial dysfunction in T cells

Effective CD8^+^ T-cell immunity in the TME depends on metabolic fitness as well as antigen recognition (19, 20). Metabolic adaptability is constrained in tumors by chronic antigen stimulation, nutrient competition, hypoxia, and suppressive metabolic signals (19, 21), so that tumor-infiltrating T cells (TILs) progressively lose durable effector function and acquire exhausted states (21). Dysfunctional TILs exhibit impaired mitochondrial membrane potential, altered mitochondrial dynamics, defective quality control, and reduced oxidative phosphorylation (OXPHOS) capacity (19, 22), abnormalities increasingly recognized as mechanistic contributors to T-cell dysfunction rather than as consequences of exhaustion alone (22).

Exhausted T cells are not metabolically equivalent. Tpex retain metabolic plasticity and remain responsive to ICB (21, 22), and relative to terminally exhausted T cells (Tex) they show preserved mitochondrial membrane potential, respiratory reserve, and mitochondrial turnover, supported by continued reliance on oxidative metabolism (22). Tex cells accumulate dysfunctional mitochondria associated with defective removal of damaged organelles through mitophagy, despite comparable or increased mitochondrial mass in some contexts (23). Mitochondrial competence rather than abundance therefore determines whether exhausted populations retain the capacity for checkpoint-mediated reinvigoration (20, 23).

Extrinsic metabolic stress amplifies these intrinsic vulnerabilities. Lactate accumulation, extracellular acidosis, adenosine signaling, and persistent hypoxia restrict metabolic flexibility and may alter intracellular pH regulation, redox balance, substrate utilization, and mitochondrial homeostasis, translating extracellular pressure into impaired mitochondrial adaptation and progressive loss of T-cell fitness (19). The resulting dysfunction is stabilized by transcriptional and epigenetic programs induced by chronic antigen exposure and sustained metabolic stress, which limit functional reprogramming even after checkpoint blockade (21, 22). In NSCLC, the association between TCF1^+^PD-1^+^ T cells and improved responses to checkpoint blockade supports the relevance of metabolically competent Tpex (24, 25), whereas the metabolically restrictive environment of these tumors may limit maintenance and expansion of the population (19).

The lactate–mitochondrial axis is defined here as a constrained metabolic module linking extracellular lactate-associated stress to mitochondrial functional states in tumor-infiltrating T cells (4, 26) (**Figure 1**). It is a dynamic interaction between extracellular pressure and intracellular adaptation rather than a single linear pathway, and it can be examined through paired measurement of extracellular stress, including lactate exposure and adenosine-associated signaling, and of intracellular parameters, including pH regulation, NAD^+^/NADH balance, mitochondrial membrane potential (ΔΨm), OXPHOS capacity, mitochondrial mass, and quality-control pathways (19). Only a subset of these measurements is currently feasible in patient-derived samples, and no single parameter represents the axis fully. The relationship may also be bidirectional, since mitochondrial dysfunction can itself promote HIF-1α-associated glycolytic reprogramming and progression toward terminal exhaustion (22), so causal direction should be established through targeted perturbation of each component.

**Figure 1:**
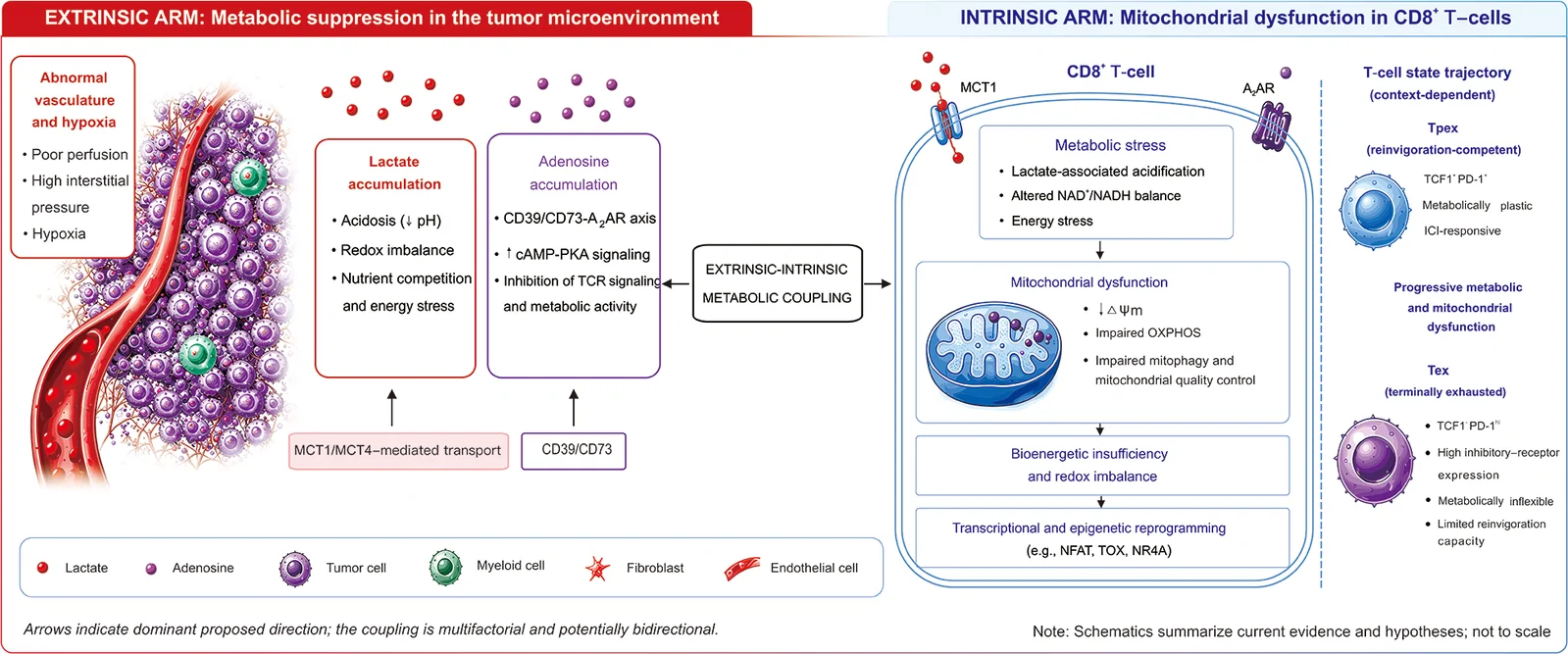
Proposed lactate–mitochondrial axis in the tumor microenvironment. Extrinsic metabolic suppression driven by hypoxia, lactate, and adenosine is proposed to contribute to mitochondrial dysfunction and exhaustion-state transitions in CD8^+^ T cells.

One tractable directional pathway involves the effect of extracellular lactate-associated stress on mitochondrial homeostasis. Elevated lactate exposure may contribute to intracellular acidification and redox imbalance, which can impair mitochondrial fitness by destabilizing membrane potential and disrupting mitochondrial maintenance (26, 27). Mitophagy is a candidate mechanistic link between metabolic stress and T-cell fate. The PINK1/Parkin pathway contributes to clearance of damaged mitochondria, impaired clearance may permit accumulation of dysfunctional organelles, and receptor-mediated pathways such as BNIP3/NIX may be engaged under hypoxia (28). Mitochondrial dynamics add a further layer, since the balance between fission and fusion, governed by proteins including DRP1, MFN2, and OPA1, influences mitochondrial distribution, respiratory efficiency, and metabolic adaptability (23), and its disruption may facilitate transition from metabolically flexible Tpex toward terminally dysfunctional states (22, 23). Other stress-response pathways, such as the mitochondrial unfolded protein response, are candidate readouts whose roles in tumor-infiltrating T cells remain insufficiently characterized (29). Which quality-control pathways predominate in human NSCLC TILs, and whether lactate-associated stress directly alters them, remain unresolved.

The relationship between extracellular acidosis and T-cell state is not uniformly detrimental. Prolonged acidosis has been reported to restrict methionine and one-carbon metabolism and to preserve stem-like features and mitochondrial respiratory capacity (30), although these observations derive largely from *in vitro* conditioning and adoptive-transfer models and do not show that endogenous Tpex are protected within acidic tumors. The consequences of acid exposure depend on differentiation state, exposure duration, and metabolic context, so extracellular acidity should not be treated as a unidirectional surrogate for T-cell dysfunction.

Two predictions follow. Reducing extracellular metabolic stress should partially restore mitochondrial fitness in metabolically competent T cells, and perturbing mitochondrial quality control should alter sensitivity to lactate-associated stress (26, 31). Whether mild hyperthermia influences these pathways is unknown, so T-cell-intrinsic adaptation is treated as an extension and not as a prerequisite. The central hypothesis concerns the extracellular arm, namely that hyperthermia may transiently modulate metabolic constraints and thereby create a more permissive environment for checkpoint-mediated reinvigoration.

## 4 Hyperthermia as a metabolic gradient modulator

Throughout this article, hyperthermia refers to locoregional mild hyperthermia; systemic or whole-body approaches involve distinct physiological effects and clinical considerations (32, 33). Within the lactate–mitochondrial axis framework, mild hyperthermia is conceptualized as a transient modulator of metabolic gradients and not as a direct immune activator. The strongest support for this view comes from tissue-level physiological effects, namely increased perfusion and improved oxygen delivery, whereas direct T-cell-intrinsic metabolic reprogramming remains speculative (8, 32) (**Figure 2**).

**Figure 2:**
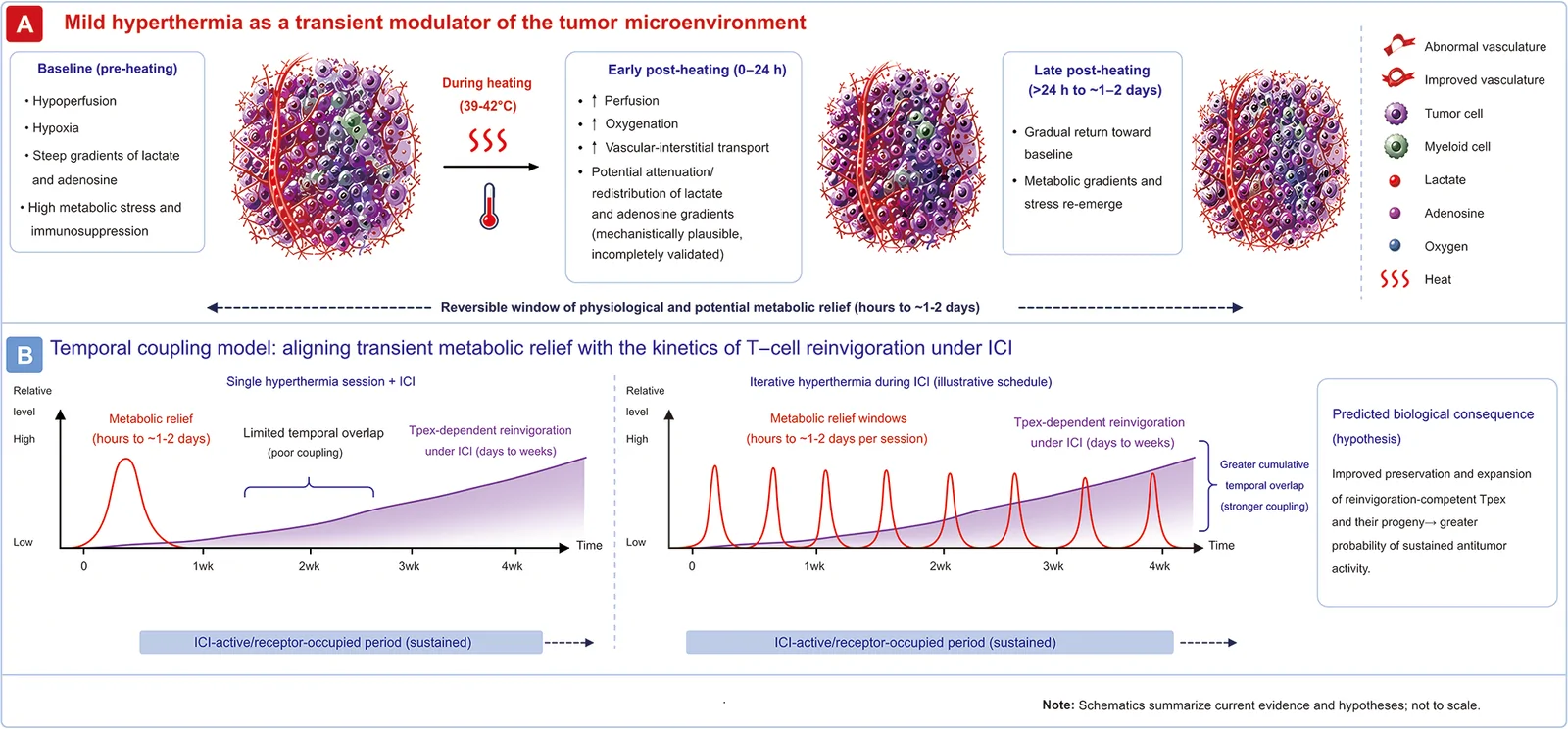
Proposed temporal-coupling model for mild hyperthermia and immune checkpoint inhibition. **(A)** Mild hyperthermia may generate transient physiological and candidate metabolic-relief windows. **(B)** Repeated sessions are hypothesized to increase the temporal overlap of these windows with ICI-driven T-cell reinvigoration, operationalized as coverage of the reinvigoration phase and, at matched coverage, the placement of relief windows within it. The illustrated schedule in **(B)** is conceptual rather than a validated clinical regimen; ICI is administered according to its standard regimen, with hyperthermia sessions distributed within the ICI dosing interval rather than timed against individual infusions.

### 4.1 Evidence-supported tissue-level effects

At 39–42 °C, mild hyperthermia acts predominantly as a physiological rather than a cytotoxic intervention. Thermal exposure induces vasodilation and is generally associated with increased local blood flow, reduced perfusion heterogeneity, and improved oxygen delivery to hypoxic tumor regions (8, 32). Across preclinical models these changes have been reported to persist from minutes to approximately 1–2 days depending on tumor type, heating conditions, and thermal stress duration (8). The lower end of this range is not a theoretical extreme. In a murine mammary carcinoma, tumor oxygenation returned to pre-heating values within minutes of ending a single mild-hyperthermia exposure, whereas elevation persisted up to 24 h in other models measured in the same review (8). Relief-window duration therefore cannot be assumed from the literature and must be measured in each setting, with direct consequences both for the sampling density needed to detect a window and for the coverage estimates.

At higher thermal doses, vascular damage can produce paradoxical perfusion shutdown, and the upper boundary is species- and tumor-dependent (8, 32). It is commonly placed near 43 °C on the basis of rodent tumor models, whereas in spontaneous canine soft tissue sarcomas oxygenation improved overall after hyperthermia, with the largest gains at median treatment temperatures (T50) below approximately 44 °C and reduced pO_2_ with an increased hypoxic fraction above that level (34). The boundary should be verified by thermometry in individual patients (32).

By transiently improving perfusion, hyperthermia is expected to enhance convective transport and vascular–interstitial exchange, increasing the mobility of soluble metabolites and influencing the spatial distribution of lactate and adenosine (8, 32). Direct *in vivo* evidence for net intratumoral depletion remains limited, so such changes more probably reflect redistribution and gradient attenuation than depletion. Redistribution is not by itself relief, because whether it reduces suppressive exposure at the sites where T cells reside is a separate question, and it is that site-specific form of redistribution that the operational criteria of Section 4.3.1 record (**Table 1**).

**Table 1:** Testable predictions and falsification criteria of the heat–metabolism–immunity framework.

Component	Testable prediction	Test/readout	Expected result	Result that would challenge it
Physiological relief (Level 1)	Transient increase in tumor perfusion and oxygenation	DCE-MRI; BOLD/TOLD MRI^d^; hypoxia-sensitive PET where available	Change > RC, in the direction of improved oxygenation, for ≥1 imaging interval	No change, or change < threshold^a^; perfusion-only response
Candidate lactate-associated gradient modulation (Level 2)	Hypoxia attenuation with lactate/adenosine redistribution away from TIL-dense regions; bulk acid–base direction not predicted^e^	Hyperpolarized ¹³C-pyruvate MRI ratio (TIL-dense vs tumor-wide signal); biopsy ectonucleotidase/adenosine-pathway markers (lymphocyte-dense regions)	Decrease > RC in the prespecified lactate-related regional/tumor-wide signal ratio, without a rise in regional absolute signal; adenosine-pathway change supportive where tissue is available	Ratio unchanged/rising despite improved perfusion; or fall with rising regional signal
Repeatability across sessions	Relief windows regenerate across serial sessions; amplitude/phase may vary^c^	Serial DCE-MRI/BOLD-TOLD, sessions 1–3 and maintenance; CEM43T90 where feasible^b^	Parameters ≥ threshold^a^ at sessions 2–n	Decline below threshold^a^ despite reproducible delivery (non-repeatable relief)
Reinvigoration-competent T-cell compartment (Level 3)	Candidate relief is immunologically relevant primarily when a checkpoint-responsive Tpex compartment is present	Paired biopsy; flow cytometry of TCF1^+^PD-1^+^CD8^+^ T cells	Relief-window exposure is associated with subsequent Tpex proliferation/effector differentiation, preferentially in tumors with a measurable baseline Tpex compartment	Comparable reinvigoration occurs despite a consistently low/absent baseline Tpex compartment
Temporal coupling (Level 3)	Reinvigoration is associated with candidate-window coverage and, conditional on comparable coverage, differs according to prespecified placement within the estimated reinvigoration phase^g^	Randomized placement vs ICI initiation; window onset/duration; serial Tpex, TCR sequencing	Reinvigoration is associated with achieved coverage and, at comparable coverage, differs by placement	Reinvigoration independent of coverage/placement; or tracks session count alone
Scope restriction (deferred)^i^	Framework applies mainly to metabolically-suppressed, Tpex-competent tumors	Baseline hypoxic subvolume and liver-normalized SULpeak, analyzed together with TCF1^+^PD-1^+^CD8^+^ frequency as continuous covariates	Reinvigoration, and at the efficacy stage response, increase with baseline metabolic suppression and with baseline Tpex frequency	No gradient across the observed range of either covariate
Tumor-growth monitoring and safety	Repeated heating does not accelerate growth/dissemination of heated lesion(s)^h^	Serial cross-sectional measurement vs unheated reference lesions	Heated-lesion growth comparable to reference/expected trajectory	Heated lesion progresses faster → triggers stopping rule
Mitochondrial adaptation	Hyperthermia improves T-cell mitochondrial fitness^f^	ΔΨm, mitochondrial mass; OXPHOS (e.g., SCENITH); PINK1/Parkin; mitophagy flux	Improved mitochondrial readouts in plastic TIL subsets	Relief without mitochondrial change (narrows, does not refute)
Local-to-systemic transfer	Local heating + systemic ICI affects unheated lesions^f^	TCR clonality (tumor/blood); multi- vs single-lesion imaging	Shared expanded clones across sites; abscopal-like response	Effects confined to heated lesion(s); no local-relief link

Predictions are biological/mechanistic endpoints, not clinical-efficacy outcomes. Rows 1–6 are core predictions; row 7 is a safety/stopping-rule criterion; rows 8–9 are optional extensions (footnote f). The single-exposure feasibility gate that precedes these predictions (Phase 0, Section 4.3.3) is a trial-management rule and is given in **Table 2**, not here. Levels 1–3 refer to the evidential levels defined in Section 4.3.1 and are shown only for predictions that map directly onto those levels; repeatability and scope restriction are cross-cutting requirements rather than separate evidential levels.

^a^RC (repeatability coefficient) of each readout, estimated from paired baseline scans; the estimation rule, not the value, is prespecified per protocol (Section 4.3.1; **Supplementary Methods S1**).

^b^Delivery descriptors (power, duration, applicator, thermal modeling) used when thermometry is inadequate.

^c^Onset/peak shift alters placement and coverage (Sections 4.2, 4.3.1).

^d^BOLD/TOLD are oxygenation-sensitive, not measures of absolute pO_2_.

^e^Oxygenation gain with rising bulk acidity is compatible with oxygen unloading, not falsifying (Section 4.1). Bulk acid–base effects require a separate paired measurement using approaches such as APT, CEST, or hyperpolarized ¹³C-bicarbonate MRI, as discussed in Section 6.2.

^f^Rows 1–6 are core; a challenging result here counts against the model itself. Rows 8–9 (this footnote) are extensions; a challenging result narrows rather than refutes the framework (Sections 4.4, 4.7).

^g^Coverage/placement defined in Section 4.2; achieved coverage is mechanistic evidence, not a causal estimate.

^h^Trial safety/stopping-rule criterion (Section 4.6), monitored independent of the efficacy predictions above.

^i^Both covariates are continuous and are not dichotomized at entry (Section 6.1); a phase 0 cohort cannot support the interaction analysis this prediction requires, so it is evaluated at the efficacy stage of Section 6.5.

A competing account of the same physiological change warrants explicit consideration. Heating increases metabolic demand, and improved perfusion can increase glucose delivery as well as oxygen delivery, so glycolysis need not fall (32). Sustained exposure at 42 °C has been reported to increase lactate production across several tumor cell lines, an effect attenuated by ouabain and attributed in part to Na^+^/K^+^-ATPase-dependent modulation of glycolysis (35). Part of the oxygenation gain after mild hyperthermia has also been attributed to enhanced oxygen unloading from hemoglobin, facilitated among other factors by intensified tumor acidosis (8, 32), and local heating at 44 °C for 2 h raised tumor lactate and lowered tissue pH by approximately 0.2 units (8). Because vascular impairment can itself lower pH at such exposures, these data show that oxygenation and acid–base responses can dissociate after heating, but not that they do so at 39–42 °C. The same reasoning implies that improved delivery may serve tumor cells as well as T cells, which is why it is carried forward as a monitoring requirement in Section 4.6 rather than restated there as an argument.

Within the same tumor and session, therefore, oxygenation, metabolite exposure, and acid–base status may move independently or in opposing directions (8, 32), although improved oxygen availability has also been argued to attenuate glycolytic flux. Oxygenation is accordingly treated throughout this article as a physiological readout and not as a surrogate for metabolic relief, and the net direction of the metabolic response is a separate falsifiable component in **Table 1**. In the remainder of this article, relief refers to attenuation of hypoxia together with redistribution of lactate and adenosine away from regions occupied by infiltrating T cells. A fall in bulk extracellular acidity is excluded from this definition, because the oxygen-unloading mechanism predicts that improved oxygenation and increased acid load may coexist (8); bulk acid–base status is measured alongside the predictions of the framework and not counted among them (Section 6.2), while the predictions in **Table 1** are specified on spatially resolved readouts.

The evidence summarized here derives mainly from rodent tumors, canine soft tissue sarcomas, and human tumors outside the lung, and its endpoints are predominantly perfusion and oxygenation. Direct measurements of metabolite redistribution at T-cell-containing sites during mild hyperthermia in human NSCLC have, to our knowledge, not been reported.

### 4.2 Hypothesis: iterative metabolic relief and temporal coupling with T-cell reinvigoration

The central hypothesis is that, in glycolytic, hypoxic NSCLC retaining reinvigoration-competent T cells, the temporal alignment of recurrent candidate metabolic-relief windows with ICB-driven T-cell reinvigoration may influence the immunological contribution of mild hyperthermia, beyond the magnitude of an individual thermal response. The hypothesis does not exclude contributions from the depth or spatial extent of relief, or from the alternative mechanisms considered in Section 7. Because those physiological changes are short-lived relative to ICB-driven reinvigoration (8, 11), a single session may be insufficient once suppressive gradients re-establish. A relief window is an interval during which tumor regions containing infiltrating immune cells meet the relief definition given above, operationalized in Section 4.3.1, and the most critical unverified assumption of the model is that repeated sessions generate such windows reproducibly in human tumors.

The model rests on four linked and separately testable assumptions. First, mild hyperthermia should produce reproducible increases in tumor perfusion and oxygenation. Second, these changes should translate into reduced or redistributed exposure to immunosuppressive metabolites in immune-relevant regions. Third, the tumor should contain reinvigoration-competent T cells, particularly Tpex (7, 24). Fourth, the timing of relief windows should overlap with the phase in which ICB-induced reinvigoration occurs. Failure at any step would challenge the proposed mechanism. The framework does not propose that mild hyperthermia directly restores exhausted T-cell function; where windows occur in tumors containing metabolically competent Tpex, intermittent reduction of HIF-1α-associated glycolytic pressure might help preserve Tpex fitness or slow progression toward terminal exhaustion (22).

Reinvigoration denotes the ICB-driven transition of TCF1^+^PD-1^+^ progenitor exhausted CD8^+^ T cells toward proliferative, cytotoxic effector-like states and the clonal expansion of checkpoint-responsive clones, and not reversal of terminal exhaustion (7, 36). It would be tracked through serial Tpex quantification with Ki-67 and granzyme B and, where feasible, T-cell receptor sequencing (11, 36). The boundaries of the reinvigoration phase are not established in patients and probably vary between them, so coverage and placement are exposure measured against an estimated and uncertain phase, and a misestimated denominator can inflate or deflate either quantity independently of any effect of hyperthermia. Blood-based proliferative kinetics of PD-1^+^ CD8^+^ T cells after checkpoint blockade may offer individual-level anchors for these boundaries (37, 38), although their correspondence to intratumoral reinvigoration is not established.

Because PD-1/PD-L1 antibodies maintain prolonged receptor occupancy between doses, the interval between a thermal session and the nearest infusion may not be the biologically relevant variable (39). The reference point proposed here is the initiation of checkpoint blockade, after which precursor exhausted clones revive and expand (7, 36). Two schedule-level quantities are defined on this basis and are recorded separately at the first two evidential levels defined below, because they are not interchangeable. Physiological-window coverage is the fraction of the post-initiation reinvigoration phase during which a detected physiological window is open, and candidate metabolic-relief-window coverage is the fraction of that phase during which a window meeting both the physiological and the candidate metabolic criterion is open. The two can diverge widely. A patient whose perfusion improves after every session, but in whom metabolite-related signal in lymphocyte-dense regions does not fall, would show high physiological coverage and no candidate metabolic coverage; where metabolic readouts are absent or inadequate, metabolic coverage is recorded as not estimable and treated as missing data, without imputation from physiological coverage. Placement is the position of windows relative to the reinvigoration phase, summarized as the proportion of delivered windows falling inside it and the position of window density within it, so that windows delivered predominantly outside the phase constitute mistimed exposure. Candidate metabolic-relief-window coverage is the primary mechanistic quantity, physiological-window coverage indicates delivery quality, and placement addresses whether timing carries information beyond coverage. Coverage can be estimated only from imaged sessions, so any extrapolation of window duration to unimaged sessions is an assumption to be examined in sensitivity analyses. The prespecified schedule that determines intended placement can be randomized, whereas achieved placement and coverage are post-randomization observations, so achieved coverage is specified as a mechanistic mediator and its association with outcome is interpreted as mechanistic evidence rather than as an unconfounded causal estimate.

### 4.3 Operationalization and repeatability of the relief window

The iterative model assumes that serial sessions remain capable of generating physiologically meaningful responses. Repeated heating engages adaptive heat-shock responses that reduce cellular thermal sensitivity, but whether the tissue-level vascular response follows the same kinetics is a distinct question (32). In rodent tumors, the blood-flow response to a second exposure has been reported to exceed that after a single exposure, termed vascular thermotolerance, and inter-session intervals of approximately 2–3 days have on this basis been proposed for combination with fractionated radiotherapy (40). In canine soft tissue sarcomas treated with fractionated hyperthermia and radiotherapy, improved oxygenation persisted across the course in tumors with low pretreatment oxygenation, whereas less favorable changes were seen in others (41).

Repeated heating regimens have also been delivered outside oncology, which bears on their deliverability and tolerability rather than on their biological effect in tumors. Transrectal or transurethral microwave hyperthermia has been given to men with benign prostatic hyperplasia in five to ten sessions over several weeks, weekly to twice-weekly, with histological and serum PSA-based safety endpoints and no evidence of irreversible tissue damage in the reported series (42, 43). These regimens used local tissue temperatures around 42–43 °C, at or above the upper end of the range considered here and did not provide the vascular or metabolic readouts required by the present framework.

Repeated passive heat exposure in non-oncologic human settings has also produced vascular adaptations in some studies, but recent systematic reviews indicate substantial heterogeneity and no consistent improvement across all vascular endpoints (44, 45). These data support the plausibility of physiological adaptation to repeated heat exposure without establishing that comparable adaptation occurs in tumors. The available evidence therefore does not support a simple assumption of progressive attenuation and suggests that repeatability should be treated as an empirical property of the individual tissue response rather than as a fixed property of the intervention.

#### 4.3.1 Operational criteria and evidential levels for a relief window

No consensus threshold currently defines a biologically meaningful relief window, so provisional criteria are proposed at three evidential levels, matching the strength of each claim to the directness of the measurement behind it. At the first level, a physiological window is recorded when a prespecified perfusion or oxygenation-sensitive imaging metric changes beyond the repeatability coefficient (RC) of that readout and the change is confirmed at the next scheduled measurement (46, 47); a change present at one time point but not the next is recorded as an unconfirmed transient response with an interval-censored closure time rather than as a failure to detect a window (48). The RC is estimated from the paired baseline scans of the cohort as 2.77 times the pooled within-subject standard deviation rather than adopted from the literature, because repeatability varies substantially with acquisition and analysis method for DCE-MRI (47, 49) and for BOLD and TOLD parameters (50); it defines detectability above measurement error and not a biologically meaningful effect. Detected responses are classified as hypoxia-consistent, supported where available by hypoxia-sensitive PET (51), or as perfusion-only, and only the former enter analyses of the core mechanism. At the second level, a candidate lactate-associated metabolic-relief window is recorded when a hypoxia-consistent response co-occurs with a fall, exceeding the RC of that ratio, in the ratio of metabolite-related signal in lymphocyte-dense regions to the tumor-wide signal on hyperpolarized ¹³C-pyruvate MRI (52), with the regional absolute signal reported alongside it; biopsy-based adenosine-pathway readouts are referenced to the spread across fields in the baseline sample. The third level is mechanistic corroboration and validation, which is not defined by a threshold but by a calibration analysis from which a minimal meaningful effect may be estimated, and it is that analysis, rather than the definition of either window, that is the evidential basis of any claim that metabolic suppression has been relieved. Measurement is constrained throughout by scale since MRI voxels are coarser than the lymphocyte microenvironment and adenosine exposure cannot at present be imaged directly (53). The full definitions, the estimation and classification rules, and the four classes of negative observation are set out in **Supplementary Methods S1**; these cut-offs are provisional and illustrative rather than validated analytical procedures, and the thresholds referred to elsewhere, including those in **Table 2**, are these.

**Table 2:** Illustrative decision matrix for prospective evaluation of individualized thermal scheduling.

Baseline Tpex frequency (continuous; reporting strata only)	Early imaging response (mechanistic continuation rule)	Thermal toxicity (safety stopping rule)	Action	Recorded as
Any (value recorded)	Meets hypoxia-consistent threshold at session 1; stable/increased later	< grade 2	Maintain reference schedule; reassess vs. coverage/placement	Repeatable hypoxia-consistent window
Any (value recorded)	Meets hypoxia-consistent threshold at session 1, then declines	< grade 2	Lengthen the inter-session interval prospectively (e.g., reduce from three to two sessions/week) and reassess repeatability	Non-repeatable relief (Section 4.5)
Any	Exceeds threshold at one time point, not the next	Any	Sample more densely at next session before deciding	Unconfirmed transient response (Section 4.3.1)
Any (value recorded)	Below threshold at session 1 (adequate thermometry/sampling)	Any	Do not escalate to the iterative schedule; repeat under prespecified conditions where protocol permits, or discontinue study hyperthermia according to the phase-0 stopping rule	Failure to detect a window
Any	Below/declining threshold, inadequate thermometry	Any	Continue ICB; review delivery quality first	Technical failure, not counted against the model
Low	Any	Any	No change to the action determined by imaging response and tolerability; report within this stratum	Stratum-specific analysis
Not assessable	Any	Any	No change to the action determined by imaging response and tolerability; report as Tpex not assessable	Tpex not assessable (Section 6.1)
Any	Any	≥ grade 2, or repeated missed sessions	Hold, reduce, or discontinue study hyperthermia according to prespecified toxicity-specific rules	Safety/feasibility endpoint

The boundary defining a low baseline Tpex frequency is prespecified per protocol as an administrative label for stratified reporting and has no biological standing (Section 6.1); a sample that does not meet the counting-adequacy threshold is recorded as not assessable rather than as low. Continuation, adaptation and stopping actions in this matrix are determined by the early imaging response and by tolerability; baseline Tpex frequency is measured in every patient and its strata are used for exploratory reporting only and do not trigger a treatment decision. The imaging and tolerability rows and the Tpex reporting rows are therefore not mutually exclusive, and each patient falls within one of each. Imaging thresholds are those of Section 4.3.1 and **Supplementary Methods S1**. Session 1 above corresponds to the single test exposure of Phase 0 (Section 4.3.3); escalation and non-escalation are trial-management actions recorded here rather than as a biological prediction in **Table 1**.

#### 4.3.2 Serial evaluation of relief-window reproducibility

Serial evaluation should characterize each session with an identical set of readouts, applied at early and later sessions, for example the first three sessions and a later maintenance session. These comprise the area under the curve of the change in perfusion, assessed by dynamic contrast-enhanced (DCE) MRI, and of the change in oxygenation-sensitive signal, assessed by BOLD or TOLD MRI (47, 50), together with window duration and time to onset and peak, since placement depends on when relief begins and not only on its size. BOLD and TOLD signals do not measure absolute pO_2_, and hypoxia-sensitive PET may serve as a complementary readout where available (50, 51). These measurements are paired with the metabolic readouts defined above, are acquired at the same post-session times at every evaluated session, and the repeatability of each measurement should be established beforehand from paired baseline scans (47, 49).

Thermal exposure should be documented for every session (54). Where reliable thermometry is available, it should be summarized using spatially informative temperature metrics and cumulative equivalent minutes at 43 °C, for example CEM43T90 where technically valid (54, 55), noting that the reference temperature is a dosimetric convention and not a biological threshold (55). Where intratumoral thermometry is inadequate, delivered power, treatment duration, applicator settings, and patient-specific thermal modeling should be recorded as delivery descriptors and not treated as equivalent measures of thermal dose (55, 56). This documentation separates technical from biological failure throughout **Table 2** and Section 4.5. A decline across sessions despite technically adequate and reproducible delivery is the non-repeatable relief condition of Section 4.5; it would argue for lengthening the inter-session interval beyond the approximately 2–3 days proposed on the basis of vascular thermotolerance (40). Stability across sessions would satisfy the minimum requirement of the model. Augmentation raises achieved coverage at an unchanged frequency and does not by itself justify more frequent sessions, since coverage is jointly determined by window duration, session frequency, temporal overlap between windows, and placement within the reinvigoration phase, not simply their product; frequency should be reassessed only against achieved coverage and placement, and prospectively.

#### 4.3.3 Phase 0 within-patient validation before efficacy testing

Before efficacy-oriented trials, a phase 0-style mechanistic study should establish whether relief windows occur in patients and whether they are reproducible within individual tumors. Its purpose is limited to the first two evidential levels above, so corroboration is exploratory at this scale, validation lies beyond its scope, and clinical benefit is not an objective. The study would enroll patients with driver-negative NSCLC scheduled to receive checkpoint blockade whose lesions can be heated with reliable thermometry (56–58), which excludes central or peribronchial lesions prone to thermal cold spots, preferably in the PD-L1-high ICB-monotherapy setting for the reasons given in Section 6 (2), although this is a preferred study context rather than an eligibility criterion. Where chemo-immunotherapy is used, the first session and its readouts should precede the first chemotherapy dose. This first evaluated session serves as the single test exposure for the phase 0 assessment, generating the physiological and metabolic response used by the prespecified feasibility gate before repeated exposure is considered (**Table 2**).

Where feasible, the first session should be delivered before or at initiation of ICB, so that direct thermal effects can be characterized before substantial ICB-driven immune remodeling (36, 37) and without delaying standard treatment; where logistics prevent this, the interval between the first session and ICB initiation should be prespecified and recorded as a covariate. Two baseline scans (47, 49), and where available an unheated reference lesion, would allow heating effects to be separated from spontaneous fluctuation in tumor hypoxia and from treatment-related changes.

Sampling times should be prespecified rather than left to convenience because they determine which windows are detectable at all. The schedule, which resolves both the few-hour windows treated as plausible here and the 1–2-day responses reported in preclinical models (8) and is applied identically at every evaluated session, is given in **Supplementary Methods S2**. The immune readouts of Sections 4.2, 4.4 and 4.6 would be collected at baseline and at a later on-treatment time point, supplemented by peripheral blood monitoring because repeated biopsy of thoracic lesions carries procedural risk (59, 60). T-cell receptor sequencing is deferred to larger studies (36), so the design can address whether windows are generated and repeated but cannot confirm clonal reinvigoration. Progression to combination efficacy testing would depend on a prespecified proportion of patients meeting the physiological and candidate metabolic criteria, with the four classes of negative observation defined in **Supplementary Methods S1** reported separately.

#### 4.3.4 Implications for treatment scheduling

The optimal frequency, duration, and timing of repeated hyperthermia remain unknown, and any proposed schedule is an experimental design and not an established regimen (10, 57). One reference schedule can serve as a comparator anchor, namely sessions of approximately 60 minutes targeting an intratumoral temperature of 40–41 °C, with dose specification, thermometry, and documentation following the quality-management requirements for regional deep hyperthermia (61), the appropriate reference class for thoracic lesions (62); the requirements for superficial hyperthermia apply only to an accessible chest-wall lesion. Sessions would be delivered two to three times per week during the first 2–4 weeks of checkpoint blockade and weekly thereafter, in line with hyperthermia–radiotherapy protocols (40, 63), and distributed within the antibody dosing interval rather than timed against it (39).

The reference frequency follows from the window durations of Section 4.1. If windows persist on the order of a day, two to three sessions per week could cover a substantial part of the reinvigoration phase; if they persist for only a few hours, as is plausible for less favorable perfusion responses and for metabolic windows specifically (8), a 6-hour window delivered three times per week covers on the order of 11% of a continuous multi-week phase. Coverage therefore depends jointly on window duration, session frequency, and individual tumor responsiveness, which should be measured and not assumed, and the sampling schedule of **Supplementary Methods S2** is what makes the short-window case distinguishable from no response. Coverage contributed by responses with a censored closure time is reported as a range, as specified in Section 4.3.1.

Decisions during a trial should follow prespecified procedures. The matrix in **Table 2** is illustrative, is not a validated decision rule, and should be reported as an exploratory element. Discontinuing thermal treatment after a first-session response below threshold despite adequate thermometry and adequate sampling is a pragmatic trial rule and not a biological conclusion, since a window not detected at the first session may still reflect the sensitivity of the imaging or the thermal parameters used, and such sessions are classified as set out in Section 4.3.1. Alternative schedules, such as a single weekly session throughout (63) or clustering across the first two ICB cycles, warrant head-to-head evaluation to determine whether schedule-dependent biological coupling exists, with completion rate and treatment-related toxicity reported as co-primary feasibility outcomes.

### 4.4 T cell-intrinsic adaptation: a testable but unvalidated extension

A more exploratory extension is that mild hyperthermia may act as a low-intensity hormetic stressor capable of engaging mitochondrial quality-control pathways, a concept established in non-thermal, non-tumor settings (64). In non-immune tissues, sublethal heat exposure has been associated with programs linked to mitochondrial biogenesis and oxidative adaptation (65). The repeated non-oncologic regimens summarized in Section 4.3 show that comparable exposures can be delivered over weeks to months, but they measured neither mitochondrial nor immune endpoints and therefore provide no support for this extension.

Direct extrapolation to the tumor immune context is constrained. Tumor-infiltrating lymphocytes are subject to chronic antigen stimulation, nutrient competition, hypoxia, and exhaustion-associated epigenetic programs that limit reversibility (66, 67), so brief thermal exposure alone is unlikely to restore mitochondrial mass, membrane potential, or oxidative capacity once dysfunction is deeply established, and current evidence does not show that mild hyperthermia improves mitochondrial fitness in tumor-infiltrating CD8^+^ T cells.

Testing this extension requires a limited set of readouts specified in advance and obtainable from small biopsies. A minimal core set is proposed in which each readout addresses a distinct component of mitochondrial competence, namely mitochondrial membrane potential and mass by flow cytometry for mitochondrial state (23); oxidative phosphorylation capacity, approximated indirectly by flow-cytometry-based functional profiling such as SCENITH, which infers metabolic dependence from the effect of inhibitors on translation rate and does not measure respiratory flux (68); proteins of the PINK1/Parkin pathway as indirect indicators of mitophagy engagement, whose steady-state levels do not report mitophagic flux (69); and the dynamics regulators DRP1, MFN2, and OPA1 for fission and fusion balance (70). Receptor-mediated mitophagy markers such as BNIP3/NIX and markers of the mitochondrial unfolded protein response such as ATF4 and CHOP (71) form an extended set, to be added where sample quantity allows or where the core set indicates a change. Mitophagy flux itself is difficult to measure in human samples and may require ex vivo systems (69).

Within patients, the core set would be measured in paired baseline and on-treatment samples and analyzed according to whether a detected physiological window and a candidate metabolic-relief window occurred, so that changes can be related to relief rather than to treatment exposure alone. The direction of effect would be addressed ex vivo, by exposing sorted tumor-infiltrating T cells to lactate or acidic conditions (26) with and without perturbation of PINK1/Parkin-dependent quality control, which tests the predictions of Section 3. Neither approach establishes causation in patients, and a failure to observe improved mitochondrial fitness would argue against this extension without refuting the tissue-level relief hypothesis.

### 4.5 Mechanistic boundaries: where the framework should and should not apply

The necessary conditions, enabling requirements, and failure conditions set out below are separately assessable, and none is proposed to be sufficient for clinical benefit. The framework is most applicable where metabolic suppression, rather than antigen insufficiency or impaired antigen presentation, is the dominant constraint on ICB responsiveness. This is more likely in tumors with pronounced glycolytic activity, hypoxic burden, and perfusion heterogeneity, features enriched in subgroups such as KRAS/STK11 co-mutant or KEAP1-altered NSCLC, in which ICB response rates have been reported to be lower in multiple cohorts despite preserved tumor mutational burden (72, 73). These subgroups are priority groups for prospective testing on the basis of metabolic phenotype and are not established beneficiary populations. High ^18^F-FDG uptake should not itself be read as evidence of metabolically mediated resistance to ICB, because whole-lesion glucose avidity is not equivalent to suppressive metabolite exposure at the sites where T cells reside and has shown context-dependent associations with response to checkpoint blockade (Section 6.1). STK11/LKB1-altered tumors in particular have been reported to show reduced T-cell infiltration (72, 73), which works against the first enabling requirement below, so whether the metabolic and immunological requirements co-occur in an individual tumor is an empirical question to be verified at baseline and not inferred from genotype. Where resistance is driven primarily by deficient antigen presentation, low tumor mutational burden, or minimal T-cell infiltration, alleviating metabolic gradients is unlikely to be rate-limiting (74).

Even when metabolic suppression is a dominant constraint, transient relief alone is unlikely to generate durable immune responses. Two enabling requirements, which are not jointly sufficient, are proposed. The first is a sufficient pool of metabolically plastic, reinvigoration-competent T cells at the time of intervention, operationally approximated by TCF1^+^PD-1^+^ progenitor exhausted CD8^+^ T cells (7, 24). The second is concurrent pharmacological attenuation of dominant inhibitory signaling, typically PD-1/PD-L1 blockade (7). Where the Tpex compartment is markedly reduced or T-cell infiltration is minimal, relief lacks an effective immune effector arm, and without checkpoint blockade even reinvigoration-competent T cells are unlikely to translate relief into sustained effector activity.

Five failure conditions warrant explicit consideration; each is defined here once and referred to by name elsewhere. The first is thermal delivery limitation, in which air interfaces and respiratory motion in central or peribronchial lung tumors generate cold spots, leaving tumor volumes below the 40–41 °C target of the reference schedule and in places below the 39 °C lower bound (56, 75). The second is paradoxical perfusion impairment, in which intratumoral temperatures above the mild range reduce and do not enhance perfusion (33). The third is temporal misalignment, in which relief windows do not overlap with phases of T-cell responsiveness. The fourth is epigenetically stabilized exhaustion, in which chronic antigen exposure has entrenched exhaustion-associated programs so that transient relief may be insufficient regardless of delivery precision (76). The fifth is non-repeatable relief, in which serial responses decline below the prespecified threshold despite technically adequate and reproducible delivery, which differs from the third condition in that the windows there are intact but mistimed. None of the five is established by a session that is not evaluable, or by an unconfirmed transient response, as those terms are defined above. Each condition is in principle prospectively identifiable, and the falsifiable predictions associated with each component are summarized in **Table 1**. The value of the framework will depend on whether its predictions hold within its defined scope and not on its ability to account for all forms of immunotherapy resistance.

### 4.6 Potential adverse biological consequences of repeated hyperthermia

Repeated heating may carry consequences that the relief model does not capture. Cumulative effects on normal tissue in the energy path, including fibrotic remodeling of the chest wall and adjacent lung parenchyma, have not been characterized for thoracic targets heated repeatedly alongside checkpoint blockade, and temperatures above the mild range can damage the vasculature and impair perfusion (77). A retrospective series of repeated chest-wall re-irradiation combined with hyperthermia, conducted in recurrent breast cancer rather than in lung parenchyma, reports low but non-zero rates of late grade 3–4 toxicity, providing an empirical, if indirect, estimate of this risk (78). Thermotolerance develops in both tumor and normal tissue after heat exposure, with kinetics that differ between the two, so selection for thermotolerant tumor cells through heat-shock protein programs is a possibility that the framework should monitor rather than assume away (79).

Whether repeated thermal stress accelerates T-cell exhaustion through chronic stress signaling has not been examined in this setting. Because serial Tpex sampling is already required by the phase 0 design, this possibility can be monitored on the same samples rather than left as a theoretical concern. The proposed readout is the ratio of TCF1^+^PD-1^+^ progenitor exhausted cells to terminally exhausted CD8^+^ T cells, measured with exhaustion-associated transcription factors and co-expressed inhibitory receptors, for example TOX with TIM-3 and LAG-3, at baseline and at the later on-treatment time point (80). A progressive fall in the ratio or rising inhibitory-receptor co-expression across sessions in patients in whom relief windows were detected, would argue that repeated heating accelerates exhaustion and is recorded as a safety signal (**Table 1**). The comparison is informative only against the trajectory expected under checkpoint blockade alone, so unheated reference lesions and, where feasible, patients receiving checkpoint blockade without heating are required for its interpretation (80).

The same perfusion and oxygenation gains proposed to benefit T cells may also benefit tumor cells, for the reasons set out in Section 4.1 (77). Whether heating influences tumor dissemination is unresolved, since increases in perfusion and vascular permeability could in principle facilitate release of tumor cells into the circulation, and we are not aware of data on whether repeated mild heating alters the growth or metastatic behavior of NSCLC. Early trials should therefore incorporate prespecified monitoring, comprising tumor growth kinetics on the same imaging used to detect relief windows, prospectively graded thermal and immune-related toxicity, and stopping rules for patients whose heated lesions progress faster than unheated reference lesions or than expected under checkpoint blockade alone.

### 4.7 Local-to-systemic immune propagation: an optional hypothesis

Clinically applicable hyperthermia is predominantly locoregional, whereas metastatic NSCLC typically involves multiple anatomically distinct lesions, so whether metabolic relief in a heated lesion can influence unheated lesions is a legitimate question. It is not part of the core mechanism, which concerns only the local relation between relief windows and checkpoint-mediated reinvigoration, and it is not tested in the phase 0 design.

One route has three steps, none of which is currently supported by direct evidence in this setting. First, relief windows within a heated lesion help preserve metabolically competent Tpex. Second, these cells undergo clonal expansion during checkpoint blockade. Third, expanded clones or their progeny act at unheated sites. The route resembles the systemic responses sometimes seen after radiotherapy combined with checkpoint inhibition, although the driver proposed here would be metabolic relief rather than immunogenic cell death (81).

The framework does not commit to a single mechanism for the third step, and three candidate routes can be specified closely enough to be distinguished experimentally. The first is egress from the heated lesion followed by trafficking to distant sites, which would depend on sphingosine-1-phosphate receptor 1-mediated tissue exit (82) and on chemokine-receptor-guided recruitment, for which CXCR3 signaling is required as effector T cells cross tumor vascular checkpoints (83). CXCR3 activity within the tumor is also required for the response to PD-1 blockade, although in that setting it was not required for CD8^+^ T-cell entry into the tumor, so the trafficking and intratumoral roles of this receptor should be measured separately (84). The second does not require cells to leave the heated lesion, since progenitor exhausted cells residing in tumor-draining lymph nodes can supply the expanding effector pool during checkpoint blockade; on this route local relief would act by increasing the antigenic or cellular input to the draining node rather than by exporting effector cells (85). The third is systemic recirculation of progenitor-like cells, which would predict shared clones across heated lesions, unheated lesions, and blood (85). These routes differ in the extent of heating they require. Draining-node supply and systemic recirculation are compatible with heating a single lesion, whereas a requirement for local egress and re-entry at each metastatic site would favor multi-lesion heating.

The question becomes testable only after relief windows have been shown to occur locally, and the readouts that would separate the three routes are given in **Table 1**. A distant response without evidence of local relief would not support this hypothesis and would instead point toward the alternative mechanisms discussed in Section 7.

## 5 Comparison with current metabolic interventions

Current metabolic strategies generally target discrete molecular nodes. The tissue-level modulation proposed in Section 4 differs from each in scope and timescale, and the comparison clarifies what a gradient-level intervention would add.

Node-directed interventions illustrate both the potential and the limitations of pathway-specific approaches. Inhibition of lactate dehydrogenase A can reduce lactate production and improve antitumor immune activity in preclinical models, but translation remains challenging because tumor cells are metabolically plastic and activated immune cells also rely on glycolysis for specific functional states (4). Among monocarboxylate transporters, MCT4 inhibition may reduce lactate export from highly glycolytic tumor cells, and MCT1 inhibition may influence lactate uptake in oxidative populations, but expression across tumor and immune-cell populations complicates selectivity (4). Adenosine-pathway inhibition faces a comparable constraint. Clinical development of CD73- or adenosine-pathway-directed therapies has supported the targeting of metabolic immune checkpoints, although responses may depend on tumor context, alternative routes of adenosine generation, and adaptive pathway changes (86). These approaches each target one component of lactate or adenosine metabolism, whereas the proposed axis emphasizes the interaction of those components with intracellular mitochondrial adaptation.

Vascular normalization offers a third comparison, and the contrast is temporal. VEGF-directed therapies remodel tumor vasculature structurally and may produce a normalization window lasting days to weeks, which can improve perfusion, oxygenation, and immune-cell access (13), whereas mild hyperthermia induces episodic, reversible functional changes on a much faster timescale (8). Hyperthermia is therefore a distinct strategy rather than a substitute for vascular normalization, and the distinction matters in NSCLC, where heterogeneous perfusion and hypoxia contribute to metabolic immune suppression (13, 87).

Strategies aimed at restoring mitochondrial function act directly on the intracellular arm of the axis. Enhancing mitochondrial biogenesis or quality control may theoretically restore metabolic competence in dysfunctional T cells but may be limited if extracellular metabolic stress remains unresolved, whereas modifying the extracellular environment alone may be insufficient where mitochondrial dysfunction and exhaustion-associated programs are deeply established (23, 87). Extracellular metabolic pressure and intracellular mitochondrial adaptation are interacting constraints and not independent targets (23, 87).

## 6 Clinical translation: combination strategies and biomarker development

Clinical translation should evaluate achieved window coverage and placement as defined in Section 4.2, rather than session number alone. An immediate design question is whether to test the framework alongside platinum-based chemo-immunotherapy or in a chemo-free setting. In driver-negative NSCLC, durable benefit from chemo-immunotherapy is frequently limited by primary or acquired resistance (2, 3). Platinum-based chemotherapy exerts immunomodulatory effects, including immunogenic cell stress and enhanced antigen presentation (88), and can reduce lymphocyte availability, with peri-treatment lymphopenia associated with poorer outcomes under checkpoint blockade (89). Methodologically, platinum-induced lymphopenia alters the PD-1^+^ CD8^+^ T-cell population from which the reinvigoration-phase boundaries are anchored (37, 38, 90), potentially displacing the denominator of both coverage and placement and rendering them incomparable between patients receiving different chemotherapy backbones. Early mechanistic studies may therefore be better served by chemo-free settings, such as PD-L1-high tumors treated with ICB monotherapy (2).

The priority subgroups and the chemo-free setting answer different questions and should not be combined into a single set of eligibility criteria. KRAS/STK11 co-mutant and KEAP1-altered tumors are proposed in Section 4.5 as indicators of metabolic phenotype, and the same tumors are enriched for reduced T-cell infiltration and show PD-L1 expression patterns that do not align with monotherapy eligibility (72, 73), so requiring both a defined genotype and PD-L1-high status at entry would restrict accrual and select against the first enabling requirement of Section 4.5. Eligibility for the phase 0 study is therefore defined by the measurable conditions of the framework, namely a heatable lesion with reliable thermometry and a quantitatively defined hypoxic subvolume on baseline imaging; lesion glycolytic activity and baseline Tpex frequency are recorded quantitatively as continuous baseline descriptors rather than used as entry thresholds at this stage (Section 6.1). KRAS, STK11, and KEAP1 status is determined at entry and used as a prespecified stratification and enrichment variable, not as an inclusion criterion, with genotype-defined enrichment deferred to efficacy-oriented studies where the number of events makes subgroup comparison interpretable. PD-L1-high tumors treated with monotherapy are not representative of the wider driver-negative population, and the phase 0 study is designed to determine whether relief windows occur rather than to estimate an effect size; the metabolic phenotype of every enrolled lesion is recorded quantitatively at baseline so that the representativeness of the tested population can be assessed rather than assumed when the framework is extended to the chemo-immunotherapy setting.

Given the evidence boundaries noted in Section 4.1, current evidence derives mainly from preclinical studies, exploratory clinical observations, and non-NSCLC settings (10), so application in driver-negative NSCLC remains a hypothesis-driven translational strategy, justified for investigation by the frequent co-occurrence of perfusion heterogeneity, hypoxia, and glycolytic metabolic remodeling in this population (3, 9).

### 6.1 Biomarkers for patient selection

Selection should integrate metabolic and immune characteristics rather than rely on a single molecular classifier, and both requirements of Section 4.5 should be measured at baseline rather than inferred from genotype. The metabolic requirement is assessed with hypoxia-sensitive imaging, supported by ^18^F-FDG PET/CT and perfusion-based imaging, and the immune requirement with baseline quantification of TCF1^+^PD-1^+^ progenitor exhausted CD8^+^ T cells, which has been linked to ICB responsiveness in NSCLC (24, 50, 51). The measurement convention for each readout, and whether it carries a threshold at all, are given below. Histological subtype and vascular architecture may influence heat distribution and can be recorded as stratification factors.

Selection must be reproducible across sites, which requires that each readout be either bounded by a prespecified number or expressed in a form that does not depend on one. Glycolytic activity is measured on the lesion to be heated and expressed relative to a within-patient reference rather than as an absolute SUV, because SUV is not comparable across scanners, reconstruction settings, and uptake times, and is itself hypoxia-dependent (4, 18). The lesion is required to exceed the PERCIST 1.0 measurability threshold, a lesion SULpeak of at least 1.5 times the mean SUL of normal liver plus two standard deviations of that measurement (91), with the liver reference and its permitted variation between the paired baseline scans specified in **Supplementary Methods S1**. This is a measurability and quality-control requirement, not a metabolic-phenotype threshold, and beyond it liver-normalized SULpeak is carried as a continuous variable. Published absolute SUVmax cut-offs are not adopted, because they are scanner- and cohort-dependent and have generally been optimized as predictors of outcome after checkpoint blockade rather than validated as markers of the metabolically suppressed phenotype considered here (92).

The hypoxic criterion is a subvolume of at least 1.5 mL on ^18^F-FMISO PET with a tumor-to-blood ratio of at least 1.4, its size set by measurement reliability rather than biology (51, 93), and it is this criterion, rather than glycolytic activity, that constitutes the quantitative metabolic condition for entry. The immune criterion is the frequency of TCF1^+^ cells among PD-1^+^CD8^+^ T cells on baseline biopsy by triple immunostaining, a phenotypic approximation of CD8^+^ Tpex rather than a complete lineage definition (24, 53), and is not expressed as an absolute density. Published series in NSCLC report this quantity as a frequency, and the triple-positive population is scarce: in tissue-microarray cores from 553 resected, treatment-naive stage I–III NSCLC, CD8^+^PD-1^+^TCF1^+^ cells were detected in 5% of patients (94). That figure reflects archival tissue and core-based sampling as much as biology and is not an expected prevalence in advanced disease, but it is enough to show that a cut-off in cells per square millimeter has no defensible basis and would depend strongly on the area sampled.

The low-compartment boundary is therefore approached in two steps. A minimum number of PD-1^+^CD8^+^ cells to be enumerated is prespecified as a counting-adequacy threshold, and a sample not reaching it is classified as Tpex not assessable rather than as low; the minimum is chosen so that the resulting bound is interpretable, since with n denominator cells a sample in which no TCF1^+^ event is observed remains consistent with a true frequency of up to approximately 3/n. Where the count is adequate, the frequency is reported as a continuous variable. No biologically validated boundary between a low and a not-low compartment is available, so the boundary used in **Table 2** is an administrative label prespecified per protocol for stratified reporting. All three readouts are assessed on the lesion to be heated, since tumor-wide measurement would not show them co-occurring where treatment is delivered.

Three classes of threshold are used in this article, and the class is stated for each readout, because operational specification does not require that every quantity be dichotomized. The first comprises numerical thresholds taken from an external convention: the PERCIST measurability threshold above, and the ^18^F-FMISO subvolume and tumor-to-blood ratio. The second comprises study-specific measurement thresholds generated by prespecified estimation procedures applied to baseline data, namely the repeatability coefficient and the field-level reference range for biopsy readouts defined in Section 4.3.1; these become numerical once baseline data exist, and what must be prespecified is the estimation rule and the point at which the value is locked. The counting-adequacy threshold above stands apart from both, being protocol-defined and numerical from the outset. The third comprises quantities that can be measured but for which no validated biological dichotomization exists: liver-normalized SULpeak, Tpex frequency, and the adenosine-pathway readouts of Section 4.3.1, which are reported continuously or as supportive evidence rather than converted into criteria. Which of these can be moved from the third class to the second, and at what values, is among the questions the calibration analysis of Section 4.3.1 is designed to address.

A proportion of patients will have no assessable baseline Tpex value, either because thoracic biopsy is restricted to safely accessible lesions (59, 60) or because the sample does not meet the counting-adequacy threshold above, and circulating TCF1^+^ subsets have not been shown to mirror the intratumoral compartment (25). These patients are recorded as Tpex not assessable and analyzed as a separate stratum, distinct in **Table 2** from both a low and a not-low value. They can still contribute to the physiological and candidate metabolic endpoints of Sections 4.3.1 and 4.3.2, but not to the corroboration analyses of Section 4.3.1, and the analysis plan should state this rather than impute an intratumoral value from blood.

### 6.2 Biomarkers for window detection and for mechanistic validation

Detection uses the readouts and limits already specified in Sections 4.3.1 and 4.3.2 (16, 47, 50, 52). Spatially resolved tissue pH may be approached through pH-weighted imaging such as amide proton transfer or CEST MRI, or hyperpolarized ¹³C-bicarbonate (52), which remain largely investigational. Bulk acid–base status should therefore be measured separately, where feasible, using these approaches as an accompanying variable rather than as part of the definition of metabolic relief. Because the proposed mechanism is spatial rather than tumor-wide, studies should prioritize approaches that link metabolic measurement to immune-cell localization (53).

Mechanistic validation asks whether extracellular metabolic changes are accompanied by intracellular T-cell adaptation, and draws on the immune readouts already specified, namely serial Tpex quantification (7, 24), T-cell receptor sequencing for clonal expansion of checkpoint-responsive populations (36), functional metabolic profiling such as SCENITH (68), and the mitochondrial core set of Section 4.4. These assays are exploratory and are not immediately deployable clinical biomarkers.

### 6.3 Accessible biomarkers and staged implementation

Early phase 0 and proof-of-concept studies can use intensive paired sampling to establish whether relief windows exist; subsequent studies can incorporate progressively more accessible measures. These were identified through a PubMed and Embase search to April 2026 combining non-small cell lung cancer and checkpoint blockade with the relevant biomarker and pathway terms, screened by one author without formal risk-of-bias assessment, retaining a measure where a plausible link could be stated to tumor burden, glycolytic or adenosine-pathway activity, or the T-cell response after blockade. The resulting list, given in **Table 3**, is a map of candidates rather than a ranking.

**Table 3:** Accessible candidate biomarkers and their status for the present framework.

Candidate	May report	Access	Proposed role
PD-L1 (IHC); STK11, KEAP1, KRAS status	ICB-monotherapy eligibility; metabolic phenotype of priority subgroups	Routine	PD-L1: definition of the preferred ICB-monotherapy study context; KRAS/STK11/KEAP1: prespecified stratification and enrichment; immune competence measured separately
Serum LDH	Nonspecific correlate of tumor burden and glycolysis (98)	Routine blood test	Stratification covariate
^18^F-FDG PET/CT	Liver-normalized SULpeak; glucose-uptake–related metabolic activity, associated with but not a direct measure of glycolytic flux, and hypoxia-dependent (4, 18)	Widely available at staging	Measurability gate for the heated lesion; continuous phenotype covariate, not dichotomized (Section 6.1)
CT-based radiomics	Image-derived phenotype; generalizability variable (96, 97)	Routine imaging, needs harmonization	Exploratory covariate
ctDNA dynamics	Systemic tumor burden, early molecular response (95)	Blood draw, assay-dependent	Systemic early-response covariate for context
Circulating Ki-67^+^ PD-1^+^ CD8^+^ kinetics	Timing of blood proliferative response (37, 38, 90)	Blood draw, flow cytometry	Anchor for estimating reinvigoration phase
Circulating PD-1^+^TCF1^+^ CD8^+^ subsets	Systemic progenitor-like phenotype; not shown to mirror intratumoral Tpex (25)	Blood draw, flow cytometry	Exploratory monitoring
Serum/plasma metabolomics; soluble CD73	Candidate metabolic/adenosine-pathway correlates (99)	Blood draw	Exploratory, hypothesis-generating
TCF1/PD-1/CD8 multiplex IHC (fixed tissue)	Baseline Tpex availability and spatial localization, reported as frequency among PD-1^+^CD8^+^ cells rather than as density (53)	Available biopsy	Baseline continuous covariate; exploratory stratification (Section 6.1)
Hyperpolarized ¹³C-MRI (pyruvate→lactate)	Real-time conversion; closest readout to lactate component (52)	Investigational; dedicated polarizer needed	Closest available lactate-related readout
DCE-MRI (perfusion)	Tumor perfusion (transfer constant); repeatability varies (47, 49)	MRI, requires contrast	Perfusion-sensitive half of minimum configuration (Section 6.3)
Oxygen-enhanced and BOLD/TOLD MRI	Oxygenation-sensitive signal; not absolute pO_2_ (50)	MRI, no tracer needed	Oxygenation-sensitive half of minimum configuration (Section 6.3)
Hypoxia-sensitive PET (e.g., ^18^F-FMISO)	Regional hypoxic burden, baseline and on-treatment (51)	Available at centers with tracer access	Baseline metabolic-phenotype eligibility in the proposed phase 0 design; complementary window detection

ICB, immune checkpoint blockade; Tpex, progenitor exhausted CD8^+^ T cells (TCF1^+^PD-1^+^); ctDNA, circulating tumor DNA; DCE-MRI, dynamic contrast-enhanced MRI; LDH, lactate dehydrogenase.

Three of these bear directly on the design proposed here. Circulating tumor DNA (ctDNA) is the most developed: early clearance or decline within approximately 3 to 9 weeks of initiation has been associated with radiographic response and survival in advanced NSCLC treated with PD-(L)1 blockade, with concordant results across systemic therapies in a meta-analysis of 32 studies (95). That time frame places ctDNA as a systemic early-response covariate rather than a session-level marker of relief. Proliferation of Ki-67^+^ PD-1^+^ CD8^+^ T cells appears in blood within the first weeks after PD-1 blockade, mostly after the first or second cycle, and is absent or delayed more often in patients with progression (90); among the candidates considered here, these kinetics provide the most direct anchor for the otherwise uncertain boundaries of the reinvigoration phase in an individual patient, on which coverage and placement depend. CT-based radiomics uses routinely acquired imaging, but predictive performance for 6-month progression-free survival fell from an AUC of 0.69 in discovery to 0.52 and 0.57 in external validation without image harmonization (96), whereas a delta-radiomics signature reached 0.8 on an external test set (97), so radiomic features enter as exploratory covariates. The remaining candidates, comprising serum lactate dehydrogenase (98), serum metabolomic signatures, soluble CD73, and circulating PD-1^+^TCF1^+^ CD8^+^ subsets (99), are less mature.

None of these candidates has been evaluated as a marker of relief windows, and because most respond to checkpoint blockade itself, they should be tested in the phase 0 setting against the imaging and biopsy readouts of Section 4.3.1, with comparison to unheated reference lesions and, where feasible, to patients receiving checkpoint blockade without heating. Their common limitation is that they report a correlate of tumor burden, global glycolysis, or the systemic response to blockade rather than exposure at T-cell-containing sites; only hyperpolarized ¹³C-MRI reports on the lactate component of the proposed relief window itself (52), which is why it is retained in **Table 3** despite its limited accessibility.

Accessibility also determines whether the intervention can be controlled in an individual patient. The session-level control described in Sections 4.3.2 and 4.3.4 rests on thermometry together with the readouts above, and none of the accessible candidates in **Table 3** substitutes for them. Where they are unavailable, the direction of the metabolic response in an individual tumor cannot be established, the decision rules of **Table 2** cannot be applied, and the framework is not implementable as specified; it should then be scaled down explicitly. A minimum implementable configuration comprises documented thermal delivery (54–56) together with one perfusion-sensitive readout, such as DCE-MRI, and one oxygenation-sensitive readout, such as oxygen-enhanced or BOLD MRI, with hypoxia-sensitive PET as a complementary alternative where available (50, 51). That configuration supports detection of physiological windows, and therefore physiological-window coverage, together with the delivery-quality and feasibility endpoints, but not the candidate metabolic criterion, so candidate metabolic-relief-window coverage is recorded as not estimable and treated as missing data under the rule of Section 4.2, without imputation from the physiological quantity. A study restricted to this configuration addresses delivery and physiology, cannot test the central hypothesis, and should not be reported as doing so. Repeated heating should not be delivered outside a trial on the assumption that relief occurs.

The first hyperthermia session may serve as an early functional readout. In human soft tissue sarcomas treated with preoperative radiotherapy and hyperthermia, the magnitude of oxygenation improvement after the first hyperthermia fraction was positively correlated with the extent of necrosis in the resection specimen (100). That observation predates current imaging and immune profiling, and the search described above identified no comparable analysis in NSCLC or in the checkpoint-blockade era. The most closely related contemporary evidence is thermometric rather than physiological: in a prospectively registered sarcoma cohort the temperature achieved within the reliably measured volume was higher in tumors showing a pathological response than in those without one (101), relating thermal delivery to outcome without measuring the physiological response the framework depends on. The case for revisiting the approach is therefore that the original physiological observation has never been retested with readouts that can be repeated non-invasively and related to immune endpoints, not that newer supporting data exist. The first-session response could serve as an early gate for continuing thermal treatment, with a provisional threshold to be calibrated in early studies.

### 6.4 Feasibility, tolerability, and safety

The schedules considered here add serial imaging and, where possible, paired biopsies to two or three weekly sessions, so patient burden is substantial and belongs among the study endpoints and not only among the limitations. Regional heating of thoracic targets is limited by normal tissue in the energy path, and the constraints of deep regional hyperthermia, including local pain, hot spots, superficial tissue injury, and systemic stress, make rigorous thermometry and quality assurance integral to delivery (61). Quality-assured regional deep hyperthermia is available at a limited number of centers (61, 62), and the additional requirement for serial MRI at fixed post-session times narrows the eligible sites further, so the accrual rate, the number of participating centers, and the proportion of screened patients meeting the thermometry requirement should be treated as design parameters and reported.

Toxicity data for hyperthermia derive mainly from combinations with radiotherapy or cytotoxic chemotherapy, and no comparable dataset exists for thoracic targets heated alongside checkpoint blockade or chemo-immunotherapy. The toxicity profile in this setting may differ for at least three reasons. First, established combinations are dominated by cytotoxic, field- and dose-related toxicities predictable from the physics or pharmacology of the concurrent modality, whereas checkpoint-blockade toxicity is immune-mediated, is not confined to the heated field, and can present with delayed onset and organ tropism, such as thyroid dysfunction, colitis, pneumonitis and hepatitis, that has no counterpart in the hyperthermia–radiotherapy or hyperthermia–chemotherapy literature; that literature cannot therefore be assumed to bound the toxicity of hyperthermia combined with ICI. Second, any interaction with ICI would have to act through immune potentiation, for example increased local antigen release, danger-associated molecular patterns, or altered immune-cell trafficking, rather than through additive cytotoxic damage within the heated field. Third, the trials that characterized toxicity used time-limited concurrent courses, whereas the iterative schedule proposed here delivers hyperthermia repeatedly over weeks to months alongside continuously active ICI. Whether transient hyperthermia alters the incidence or kinetics of immune-related adverse events has not been examined prospectively, and no difference from the radiotherapy or chemotherapy setting should be assumed in either direction. Changes in perfusion may also alter normal-tissue exposure to concurrent platinum (88).

Pneumonitis warrants separate treatment, since it is the one event to which both components could contribute in the same organ: lung parenchyma lies within or adjacent to the energy path for most thoracic targets, and checkpoint-blockade pneumonitis is not confined to any field, so an event during combined treatment cannot be attributed by location alone. Early trials should prespecify chest imaging within and outside the heated volume, report pneumonitis separately from other immune-related events, and prespecify grade-triggered rules for interrupting or discontinuing thermal treatment, since none yet exist for this combination.

Paired biopsies carry procedural risk in thoracic lesions, chiefly pneumothorax and pulmonary hemorrhage, with risk strongly dependent on lesion and needle-path characteristics (59), so biopsy should be restricted to lesions that are safely accessible, with imaging or peripheral sampling substituting where it is not. Early trials should report per-session completion rates, reasons for missed sessions, prospectively graded thermal and immune-related toxicity, and patient-reported burden captured with a validated instrument such as PRO-CTCAE (102), alongside the mechanistic endpoints. The prespecified monitoring of tumor growth kinetics in heated relative to unheated lesions, and the stopping rules for faster progression of a heated lesion, are set out in Section 4.6 and belong in the same safety-monitoring plan as the toxicity endpoints above, because both are read from the same imaging. A schedule that produces reproducible relief windows but cannot be delivered consistently has limited translational value.

### 6.5 Future directions

Prospective evaluation should proceed in three stages, each with a prespecified decision rule. The first is the phase 0 study of Section 4.3.3, whose progression criteria and negative-observation classes are defined there. The second is a randomized comparison of prespecified schedules, powered on achieved coverage and placement rather than on tumor response, with completion rate and treatment-related toxicity as co-primary feasibility outcomes; its purpose is to establish whether the schedule determines exposure, a precondition for interpreting any later association with outcome. The third is efficacy testing in which achieved coverage is analyzed as a mechanistic mediator (Section 4.2). The validation pathway follows the levels of Section 4.3.1 in sequence, from detection through corroboration to validation and then association with clinically meaningful outcomes. Failure to observe the intermediate events at any level, despite adequate thermal delivery and measurement sensitivity, would argue against the proposed mechanism and redirect investigation toward alternative effects of hyperthermia.

## 7 Discussion

The principal uncertainty lies in the iterative metabolic relief model of Section 4.2. To our knowledge, no *in vivo* study has quantified perfusion, metabolic gradients, mitochondrial fitness, and T-cell functional states together across repeated treatment cycles.

An observed improvement in ICB efficacy after hyperthermia would not by itself establish the proposed mechanism. Some alternative pathways share the same upstream physiological change (improved perfusion enhancing drug delivery, vascular permeability, or immune-cell trafficking); others are mechanistically distinct (heat-shock protein signaling, stress-associated antigen release, innate immune activation); and in multimodal settings benefit may also arise from chemo- and radiosensitization or abscopal-type immune effects. Conversely, benefit without a reproducible relief window would argue against the metabolic mechanism.

Heat-shock protein signaling is both a candidate alternative immunological pathway and the canonical mediator of cellular thermotolerance, which differs from the vascular thermotolerance seen in some models, where repeated heating preserved or enhanced tumor perfusion and oxygenation. Paired assessment of heat-shock responses and serial vascular readouts could show whether the two covary or dissociate across sessions.

Three translational challenges remain whichever model proves correct: thermal dosimetry in thoracic tumors, biological heterogeneity across T-cell and myeloid populations, and generalizability from preclinical and non-pulmonary evidence to the lung.

If validated within its stated boundaries, the framework would support further evaluation of mild hyperthermia as a metabolism-oriented sensitization strategy for checkpoint blockade in driver-negative NSCLC. Clinical efficacy should not be inferred until metabolic-relief windows are prospectively demonstrated.

## Data Availability

The original contributions presented in the study are included in the article/**Supplementary Material**. Further inquiries can be directed to the corresponding authors.

## References

[1] Sung H Filho AM Laversanne M Ferlay J Siegel RL Soerjomataram I . Global cancer statistics 2024: GLOBOCAN estimates of incidence and mortality worldwide for 34 cancers in 186 countries. CA Cancer J Clin. (2026) 76:e70090. doi: 10.3322/caac.70090 42417444 PMC13343830

[2] Hendriks LE Kerr KM Menis J Mok TS Nestle U Passaro A . Non-oncogene-addicted metastatic non-small-cell lung cancer: ESMO Clinical Practice Guideline for diagnosis, treatment and follow-up. Ann Oncol. (2023) 34:358–76. doi: 10.1016/j.annonc.2022.12.013 36669645

[3] Reck M Frost N Peters S Fox BA Ferrara R Savai R . Treatment of NSCLC after chemoimmunotherapy - are we making headway? Nat Rev Clin Oncol. (2025) 22:806–30. doi: 10.1038/s41571-025-01061-7 40813911

[4] De Martino M Rathmell JC Galluzzi L Vanpouille-Box C . Cancer cell metabolism and antitumour immunity. Nat Rev Immunol. (2024) 24:654–69. doi: 10.1038/s41577-024-01026-4 38649722 PMC11365797

[5] Ghiyabi E Arabameri A Charmi M . Mathematical modeling of hypoxia and adenosine to explore tumor escape mechanisms in DC-based immunotherapy. Sci Rep. (2024) 14:11387. doi: 10.1038/s41598-024-62209-6 38762567 PMC11102449

[6] Hatfield SM Kjaergaard J Lukashev D Belikoff B Schreiber TH Sethumadhavan S . Systemic oxygenation weakens the hypoxia and hypoxia inducible factor 1α-dependent and extracellular adenosine-mediated tumor protection. J Mol Med (Berl). (2014) 92:1283–92. doi: 10.1007/s00109-014-1189-3 25120128 PMC4247798

[7] Ahmed R Hofmann M Kallies A Oxenius A Philip M Wherry EJ . New insights into progenitor exhausted T cell populations. Nat Rev Immunol. (2026). doi: 10.1038/s41577-026-01328-9 42380632

[8] Vaupel P Piazena H Notter M Thomsen AR Grosu AL Scholkmann F . From localized mild hyperthermia to improved tumor oxygenation: physiological mechanisms critically involved in oncologic thermo-radio-immunotherapy. Cancers (Basel). (2023) 15:1394. doi: 10.3390/cancers15051394 36900190 PMC10000497

[9] Mascaux C Sen T Sanchez-Cespedes M Ortiz-Cuaran S Bossé Y Dammeijer F . Advances in lung cancer basic and translational research in 2025 - overview and perspectives focusing on NSCLC. J Thorac Oncol. (2025) 20:1369–91. doi: 10.1016/j.jtho.2025.05.024 40473107 PMC12216557

[10] Liu P Ye M Wu Y Wu L Lan K Wu Z . Hyperthermia combined with immune checkpoint inhibitor therapy: Synergistic sensitization and clinical outcomes. Cancer Med. (2023) 12:3201–21. doi: 10.1002/cam4.5085 PMC9939221 35908281

[11] Oliveira G Wu CJ . Dynamics and specificities of T cells in cancer immunotherapy. Nat Rev Cancer. (2023) 23:295–316. doi: 10.1038/s41568-023-00560-y 37046001 PMC10773171

[12] Guelfi S Hodivala-Dilke K Bergers G . Targeting the tumour vasculature: from vessel destruction to promotion. Nat Rev Cancer. (2024) 24:655–75. doi: 10.1038/s41568-024-00736-0 39210063

[13] Fan P Zhang N Candi E Agostini M Piacentini M Shi Y . Alleviating hypoxia to improve cancer immunotherapy. Oncogene. (2023) 42:3591–604. doi: 10.1038/s41388-023-02869-2 37884747

[14] Certo M Tsai CH Pucino V Ho PC Mauro C . Lactate modulation of immune responses in inflammatory versus tumour microenvironments. Nat Rev Immunol. (2021) 21:151–61. doi: 10.1038/s41577-020-0406-2 32839570

[15] Feng Q Liu Z Yu X Huang T Chen J Wang J . Lactate increases stemness of CD8 + T cells to augment anti-tumor immunity. Nat Commun. (2022) 13:4981. doi: 10.1038/s41467-022-32521-8 36068198 PMC9448806

[16] Rodríguez-Pampín I González-Pico L Selas A Andújar A Prieto-Díaz R Sotelo E . Targeting the adenosinergic axis in cancer immunotherapy: Insights into A(2A) and A(2B) receptors and novel clinical combination strategies. Pharmacol Rev. (2025) 77:100092. doi: 10.1016/j.pharmr.2025.100092 41101027

[17] Hatfield SM Kjaergaard J Lukashev D Schreiber TH Belikoff B Abbott R . Immunological mechanisms of the antitumor effects of supplemental oxygenation. Sci Transl Med. (2015) 7:277ra30. doi: 10.1126/scitranslmed.aaa1260 25739764 PMC4641038

[18] Demicco M Liu XZ Leithner K Fendt SM . Metabolic heterogeneity in cancer. Nat Metab. (2024) 6:18–38. doi: 10.1038/s42255-023-00963-z 38267631

[19] Viel S Vivier E Walzer T Marçais A . Targeting metabolic dysfunction of CD8 T cells and natural killer cells in cancer. Nat Rev Drug Discov. (2025) 24:190–208. doi: 10.1038/s41573-024-01098-w 39668206

[20] Steinert EM Furtado Bruza B Danchine VD Grant RA Vasan K Kharel A . Mitochondrial respiration is necessary for CD8(+) T cell proliferation and cell fate. Nat Immunol. (2025) 26:1267–74. doi: 10.1038/s41590-025-02202-x 40670617 PMC12307223

[21] Sun Q Dong C . Regulators of CD8(+) T cell exhaustion. Nat Rev Immunol. (2026) 26:129–51. doi: 10.1038/s41577-025-01221-x 41034337

[22] Wu H Zhao X Hochrein SM Eckstein M Gubert GF Knöpper K . Mitochondrial dysfunction promotes the transition of precursor to terminally exhausted T cells through HIF-1α-mediated glycolytic reprogramming. Nat Commun. (2023) 14:6858. doi: 10.1038/s41467-023-42634-3 37891230 PMC10611730

[23] Yu YR Imrichova H Wang H Chao T Xiao Z Gao M . Disturbed mitochondrial dynamics in CD8(+) TILs reinforce T cell exhaustion. Nat Immunol. (2020) 21:1540–51. doi: 10.1038/s41590-020-0793-3 33020660

[24] Koh J Kim S Woo YD Song SG Yim J Han B . TCF1(+)PD-1(+) tumour-infiltrating lymphocytes predict a favorable response and prolonged survival after immune checkpoint inhibitor therapy for non-small-cell lung cancer. Eur J Cancer. (2022) 174:10–20. doi: 10.1016/j.ejca.2022.07.004 35970031

[25] Dutta N Svensson J Saad GA Mello M Eklund EA Altinönder I . High baseline PD-1+ CD8 T Cells and TIGIT+ CD8 T Cells in circulation associated with response to PD-1 blockade in patients with non-small cell lung cancer. Cancer Immunol Immunother. (2025) 74:309. doi: 10.1007/s00262-025-04086-0 40944710 PMC12433424

[26] Peralta RM Xie B Lontos K Nieves-Rosado H Spahr K Joshi S . Dysfunction of exhausted T cells is enforced by MCT11-mediated lactate metabolism. Nat Immunol. (2024) 25:2297–307. doi: 10.1038/s41590-024-01999-3 PMC11588660 39516648

[27] Quinn WJ Jiao J TeSlaa T Stadanlick J Wang Z Wang L . Lactate Limits T Cell Proliferation via the NAD(H) Redox State. Cell Rep. (2020) 33:108500. doi: 10.1016/j.celrep.2020.108500 33326785 PMC7830708

[28] Wang Q Sun Y Li TY Auwerx J . Mitophagy in the pathogenesis and management of disease. Cell Res. (2026) 36:11–37. doi: 10.1038/s41422-025-01203-7 41486294 PMC12765899

[29] Zhang X Fan Y Tan K . A bird's eye view of mitochondrial unfolded protein response in cancer: mechanisms, progression and further applications. Cell Death Dis. (2024) 15:667. doi: 10.1038/s41419-024-07049-y 39261452 PMC11390889

[30] Cheng H Qiu Y Xu Y Chen L Ma K Tao M . Extracellular acidosis restricts one-carbon metabolism and preserves T cell stemness. Nat Metab. (2023) 5:314–30. doi: 10.1038/s42255-022-00730-6 36717749 PMC9970874

[31] Zhang R Gao F Li J Jin J Chen K Chaudhuri S . USP30 inhibition augments mitophagy to prevent T cell exhaustion. Sci Adv. (2025) 11:eadv6902. doi: 10.1126/sciadv.adv6902 40815646 PMC12356266

[32] Dewhirst MW . A translational review of hyperthermia biology. Int J Hyperthermia. (2025) 42:2447952. doi: 10.1080/02656736.2024.2447952 39799944

[33] Gorbaslieva I Quisenaerts T Bogers J Peeters M Saldien V Ysebaert D . Temperature-dependent effects of induced hyperthermia, including whole-body hyperthermia, on the hallmarks of cancer: A systematic review. Cancers (Basel). (2025) 17:3824. doi: 10.3390/cancers17233824 41375025 PMC12691179

[34] Vujaskovic Z Poulson JM Gaskin AA Thrall DE Page RL Charles HC . Temperature-dependent changes in physiologic parameters of spontaneous canine soft tissue sarcomas after combined radiotherapy and hyperthermia treatment. Int J Radiat Oncol Biol Phys. (2000) 46:179–85. doi: 10.1016/s0360-3016(99)00362-4 10656391

[35] Burdon RH Kerr SM Cutmore CM Munro J Gill V . Hyperthermia, Na+K+ATPase and lactic acid production in some human tumour cells. Br J Cancer. (1984) 49:437–45. doi: 10.1038/bjc.1984.70 6324839 PMC1976771

[36] Wang K Coutifaris P Brocks D Wang G Azar T Solis S . Combination anti-PD-1 and anti-CTLA-4 therapy generates waves of clonal responses that include progenitor-exhausted CD8(+) T cells. Cancer Cell. (2024) 42:1582–1597.e10. doi: 10.1016/j.ccell.2024.08.007 39214097 PMC11387127

[37] Gelibter A Tuosto L Asquino A Siringo M Sabatini A Zizzari IG . Anti-PD1 therapies induce an early expansion of Ki67(+)CD8(+) T cells in metastatic non-oncogene addicted NSCLC patients. Front Immunol. (2024) 15:1483182. doi: 10.3389/fimmu.2024.1483182 39744631 PMC11688303

[38] Kim KH Cho J Ku BM Koh J Sun JM Lee SH . The first-week proliferative response of peripheral blood PD-1(+)CD8(+) T cells predicts the response to anti-PD-1 therapy in solid tumors. Clin Cancer Res. (2019) 25:2144–54. doi: 10.1158/1078-0432.ccr-18-1449 30647082

[39] VerdonkJDJ OttenLS MontaseriG van den HeuvelMM SmeetsRL KoenenH . Translational modelling challenges receptor-occupancy-based dosing of PD-1 inhibitors like pembrolizumab. Br J Cancer. (2026). doi: 10.1038/s41416-026-03612-w 42701121

[40] Griffin RJ Dings RP Jamshidi-Parsian A Song CW . Mild temperature hyperthermia and radiation therapy: role of tumour vascular thermotolerance and relevant physiological factors. Int J Hyperthermia. (2010) 26:256–63. doi: 10.3109/02656730903453546 20210610 PMC2909325

[41] Thrall DE Larue SM Pruitt AF Case B Dewhirst MW . Changes in tumour oxygenation during fractionated hyperthermia and radiation therapy in spontaneous canine sarcomas. Int J Hyperthermia. (2006) 22:365–73. doi: 10.1080/02656730600836386 16891239

[42] Lindner A Siegel YI Korczak D . Serum prostate specific antigen levels during hyperthermia treatment of benign prostatic hyperplasia. J Urol. (1990) 144:1388–9. doi: 10.1016/s0022-5347(17)39749-5 1700150

[43] Montorsi F Galli L Guazzoni G Colombo R Bulfamante G Barbieri L . Transrectal microwave hyperthermia for benign prostatic hyperplasia: long-term clinical, pathological and ultrastructural patterns. J Urol. (1992) 148:321–5. doi: 10.1016/s0022-5347(17)36584-9 1378906

[44] Rodrigues S O'Connor F Morris N Chaseling G Sabapathy S Bach AJE . Passive heat therapy for cardiovascular disease: current evidence and future directions. Appl Physiol Nutr Metab. (2025) 50:1–14. doi: 10.1139/apnm-2024-0406 39819110

[45] Hamaya R Joyama Y Miyata T Fuse SI Yamane N Maruyama N . Non-acute effects of passive heating interventions on cardiometabolic risk and vascular health: systematic review and meta-analysis of randomized controlled trials. Am J Prev Cardiol. (2025) 23:101082. doi: 10.1016/j.ajpc.2025.101082 41049507 PMC12490526

[46] Pierce TT Sirlin CB Fowler KJ Buckler AJ Hall TJ Obuchowski NA . Understanding repeatability and reproducibility coefficients for quantitative imaging biomarkers. Radiology. (2025) 316:e250279. doi: 10.1148/radiol.250279 40793946 PMC12405707

[47] Shukla-Dave A Obuchowski NA Chenevert TL Jambawalikar S Schwartz LH Malyarenko D . Quantitative imaging biomarkers alliance (QIBA) recommendations for improved precision of DWI and DCE-MRI derived biomarkers in multicenter oncology trials. J Magn Reson Imaging. (2019) 49:e101–21. doi: 10.1002/jmri.26518 30451345 PMC6526078

[48] Dugué AE Pulido M Chabaud S Belin L Gal J . How to deal with interval-censored data practically while assessing the progression-free survival: A step-by-step guide using SAS and R software. Clin Cancer Res. (2016) 22:5629–35. doi: 10.1158/1078-0432.CCR-16-1017 27663596

[49] Woodall RT Sahoo P Cui Y Chen BT Shiroishi MS Lavini C . Repeatability of tumor perfusion kinetics from dynamic contrast-enhanced MRI in glioblastoma. Neuro-Oncol Adv. (2021) 3:vdab174. doi: 10.1093/noajnl/vdab174 34988454 PMC8715899

[50] O'Connor JPB Robinson SP Waterton JC . Imaging tumour hypoxia with oxygen-enhanced MRI and BOLD MRI. Br J Radiol. (2019) 92:20180642. doi: 10.1259/bjr.20180642 PMC6540855 30272998

[51] Reeves KM Song PN Angermeier A Della Manna D Li Y Wang J . (18)F-FMISO PET imaging identifies hypoxia and immunosuppressive tumor microenvironments and guides targeted evofosfamide therapy in tumors refractory to PD-1 and CTLA-4 inhibition. Clin Cancer Res. (2022) 28:327–37. doi: 10.1158/1078-0432.CCR-21-2394 PMC8776604 34615724

[52] Deen SS Rooney C Shinozaki A McGing J Grist JT Tyler DJ . Hyperpolarized carbon 13 MRI: Clinical applications and future directions in oncology. Radiol Imaging Cancer. (2023) 5:e230005. doi: 10.1148/rycan.230005 37682052 PMC10546364

[53] Jahangir CA Page DB Broeckx G Gonzalez CA Burke C Murphy C . Image-based multiplex immune profiling of cancer tissues: Translational implications. A report of the International Immuno-oncology Biomarker Working Group on Breast Cancer. J Pathol. (2024) 262:271–88. doi: 10.1002/path.6238 38230434 PMC11288342

[54] Bruggmoser G Bauchowitz S Canters R Crezee H Ehmann M Gellermann J . Quality assurance for clinical studies in regional deep hyperthermia. Strahlenther Onkol. (2011) 187:605–10. doi: 10.1007/s00066-011-1145-x 21932026

[55] van Rhoon GC . Is CEM43 still a relevant thermal dose parameter for hyperthermia treatment monitoring? Int J Hyperthermia. (2016) 32:50–62. doi: 10.3109/02656736.2015.1114153 26758036

[56] Gavazzi S van Lier A Zachiu C Jansen E Lagendijk JJW Stalpers LJA . Advanced patient-specific hyperthermia treatment planning. Int J Hyperthermia. (2020) 37:992–1007. doi: 10.1080/02656736.2020.1806361 32806979

[57] Ademaj A Veltsista DP Ghadjar P Marder D Oberacker E Ott OJ . Clinical evidence for thermometric parameters to guide hyperthermia treatment. Cancers (Basel). (2022) 14:625. doi: 10.3390/cancers14030625 35158893 PMC8833668

[58] Kim K Narsinh K Ozhinsky E . Technical advances in motion-robust MR thermometry. Magn Reson Med. (2024) 92:15–27. doi: 10.1002/mrm.30057 38501903 PMC11132643

[59] Saggiante L Biondetti P Lanza C Carriero S Ascenti V Piacentino F . Computed-tomography-guided lung biopsy: A practice-oriented document on techniques and principles and a review of the literature. Diagnostics (Basel). (2024) 14:1089. doi: 10.3390/diagnostics14111089 38893616 PMC11171640

[60] Huo YR Chan MV Habib AR Lui I Ridley L . Pneumothorax rates in CT-guided lung biopsies: A comprehensive systematic review and meta-analysis of risk factors. Br J Radiol. (2020) 93:20190866. doi: 10.1259/bjr.20190866 31860329 PMC7362905

[61] Bruggmoser G Bauchowitz S Canters R Crezee H Ehmann M Gellermann J . Guideline for the clinical application, documentation and analysis of clinical studies for regional deep hyperthermia: Quality management in regional deep hyperthermia. Strahlenther Onkol. (2012) 188:198–211. doi: 10.1007/s00066-012-0176-2 22926657

[62] Trefná HD Crezee H Schmidt M Marder D Lamprecht U Ehmann M . Quality assurance guidelines for superficial hyperthermia clinical trials: I. Clinical requirements. Int J Hyperthermia. (2017) 33:471–82. doi: 10.1080/02656736.2016.1277791 28049386

[63] Thomsen AR Sahlmann J Bronsert P Schilling O Poensgen F May AM . Protocol of the HISTOTHERM study: Assessing the response to hyperthermia and hypofractionated radiotherapy in recurrent breast cancer. Front Oncol. (2023) 13:1275222. doi: 10.3389/fonc.2023.1275222 38169879 PMC10759986

[64] Cheng YW Liu J Finkel T . Mitohormesis. Cell Metab. (2023) 35:1872–86. doi: 10.1016/j.cmet.2023.10.011 37939657 PMC10632604

[65] Marchant ED Nelson WB Hyldahl RD Gifford JR Hancock CR . Passive heat stress induces mitochondrial adaptations in skeletal muscle. Int J Hyperthermia. (2023) 40:2205066. doi: 10.1080/02656736.2023.2205066 37106474

[66] Scharping NE Rivadeneira DB Menk AV Vignali PDA Ford BR Rittenhouse NL . Mitochondrial stress induced by continuous stimulation under hypoxia rapidly drives T cell exhaustion. Nat Immunol. (2021) 22:205–15. doi: 10.1038/s41590-020-00834-9 33398183 PMC7971090

[67] Philip M Schietinger A . CD8(+) T cell differentiation and dysfunction in cancer. Nat Rev Immunol. (2022) 22:209–23. doi: 10.1038/s41577-021-00574-3 PMC9792152 34253904

[68] Argüello RJ Combes AJ Char R Gigan JP Baaziz AI Bousiquot E . SCENITH: A flow cytometry-based method to functionally profile energy metabolism with single-cell resolution. Cell Metab. (2020) 32:1063–1075.e7. doi: 10.1016/j.cmet.2020.11.007 PMC8407169 33264598

[69] Klionsky DJ Abdel-Aziz AK Abdelfatah S Abdellatif M Abdoli A Abel S . Guidelines for the use and interpretation of assays for monitoring autophagy (4th edition)(1). Autophagy. (2021) 17:1–382. doi: 10.1080/15548627.2020.1797280 33634751 PMC7996087

[70] Buck MD O'Sullivan D Klein Geltink RI Curtis JD Chang CH Sanin DE . Mitochondrial dynamics controls T cell fate through metabolic programming. Cell. (2016) 166:63–76. doi: 10.1016/j.cell.2016.05.035 27293185 PMC4974356

[71] Quirós PM Prado MA Zamboni N D'Amico D Williams RW Finley D . Multi-omics analysis identifies ATF4 as a key regulator of the mitochondrial stress response in mammals. J Cell Biol. (2017) 216:2027–45. doi: 10.1083/jcb.201702058 PMC5496626 28566324

[72] Skoulidis F Araujo HA Do MT Qian Y Sun X Galan-Cobo A . CTLA4 blockade abrogates KEAP1/STK11-related resistance to PD-(L)1 inhibitors. Nature. (2024) 635:462–71. doi: 10.1038/s41586-024-07943-7 39385035 PMC11560846

[73] Minor AC Couser E Eichner LJ . Targeting LKB1/STK11-mutant cancer: Distinct metabolism, microenvironment, and therapeutic resistance. Trends Pharmacol Sci. (2025) 46:722–37. doi: 10.1016/j.tips.2025.06.008 40701847 PMC12372497

[74] van de Haar J Mankor JM Hummelink K Monkhorst K Smit EF Wessels LFA . Combining genomic biomarkers to guide immunotherapy in non-small cell lung cancer. Clin Cancer Res. (2024) 30:1307–18. doi: 10.1158/1078-0432.ccr-23-4027 38300729 PMC10982639

[75] Abrishami Kashani M Campbell-Washburn AE Murphy MC Catalano OA McDermott S Fintelmann FJ . Magnetic resonance imaging for guidance and follow-up of thoracic needle biopsies and thermal ablations. J Thorac Imaging. (2022) 37:201–16. doi: 10.1097/rti.0000000000000651 35426857 PMC10441002

[76] Kang TG Johnson JT Zebley CC Youngblood B . Epigenetic regulation of T cell exhaustion in cancer. Nat Rev Cancer. (2026) 26:46–61. doi: 10.1038/s41568-025-00883-y PMC13203999 41145849

[77] Dewhirst MW Lee CT Ashcraft KA . The future of biology in driving the field of hyperthermia. Int J Hyperthermia. (2016) 32:4–13. doi: 10.3109/02656736.2015.1091093 26850697

[78] Bakker A Tello Valverde CP van Tienhoven G Kolff MW Kok HP Slotman BJ . Post-operative re-irradiation with hyperthermia in locoregional breast cancer recurrence: Temperature matters. Radiother Oncol. (2022) 167:149–57. doi: 10.1016/j.radonc.2021.12.036 34973278

[79] Urano M . Kinetics of thermotolerance in normal and tumor tissues: A review. Cancer Res. (1986) 46:474–82. 3510074

[80] Gebhardt T Park SL Parish IA . Stem-like exhausted and memory CD8(+) T cells in cancer. Nat Rev Cancer. (2023) 23:780–98. doi: 10.1038/s41568-023-00615-0 37821656

[81] Theelen W Peulen HMU Lalezari F van der Noort V de Vries JF Aerts J . Effect of pembrolizumab after stereotactic body radiotherapy vs pembrolizumab alone on tumor response in patients with advanced non-small cell lung cancer: Results of the PEMBRO-RT phase 2 randomized clinical trial. JAMA Oncol. (2019) 5:1276–82. doi: 10.1001/jamaoncol.2019.1478 31294749 PMC6624814

[82] Baeyens A Bracero S Chaluvadi VS Khodadadi-Jamayran A Cammer M Schwab SR . Monocyte-derived S1P in the lymph node regulates immune responses. Nature. (2021) 592:290–5. doi: 10.1038/s41586-021-03227-6 33658712 PMC8475585

[83] Mikucki ME Fisher DT Matsuzaki J Skitzki JJ Gaulin NB Muhitch JB . Non-redundant requirement for CXCR3 signalling during tumoricidal T-cell trafficking across tumour vascular checkpoints. Nat Commun. (2015) 6:7458. doi: 10.1038/ncomms8458 26109379 PMC4605273

[84] Chow MT Ozga AJ Servis RL Frederick DT Lo JA Fisher DE . Intratumoral activity of the CXCR3 chemokine system is required for the efficacy of anti-PD-1 therapy. Immunity. (2019) 50:1498–1512.e5. doi: 10.1016/j.immuni.2019.04.010 31097342 PMC6527362

[85] Wijesinghe SKM Rausch L Gabriel SS Galletti G De Luca M Qin L . Lymph-node-derived stem-like but not tumor-tissue-resident CD8(+) T cells fuel anticancer immunity. Nat Immunol. (2025) 26:1367–83. doi: 10.1038/s41590-025-02219-2 40730900

[86] Bendell J LoRusso P Overman M Noonan AM Kim DW Strickler JH . First-in-human study of oleclumab, a potent, selective anti-CD73 monoclonal antibody, alone or in combination with durvalumab in patients with advanced solid tumors. Cancer Immunol Immunother. (2023) 72:2443–58. doi: 10.1007/s00262-023-03430-6 37016126 PMC10264501

[87] Baessler A Vignali DAA . T cell exhaustion. Annu Rev Immunol. (2024) 42:179–206. doi: 10.1146/annurev-immunol-090222-110914 38166256

[88] Galluzzi L Humeau J Buqué A Zitvogel L Kroemer G . Immunostimulation with chemotherapy in the era of immune checkpoint inhibitors. Nat Rev Clin Oncol. (2020) 17:725–41. doi: 10.1038/s41571-020-0413-z 32760014

[89] Cho Y Park S Byun HK Lee CG Cho J Hong MH . Impact of treatment-related lymphopenia on immunotherapy for advanced non-small cell lung cancer. Int J Radiat Oncol Biol Phys. (2019) 105:1065–73. doi: 10.1016/j.ijrobp.2019.08.047 31476418

[90] Kamphorst AO Pillai RN Yang S Nasti TH Akondy RS Wieland A . Proliferation of PD-1+ CD8 T cells in peripheral blood after PD-1-targeted therapy in lung cancer patients. Proc Natl Acad Sci USA. (2017) 114:4993–8. doi: 10.1073/pnas.1705327114 28446615 PMC5441721

[91] Wahl RL Jacene H Kasamon Y Lodge MA . From RECIST to PERCIST: Evolving considerations for PET response criteria in solid tumors. J Nucl Med. (2009) 50:122S–50S. doi: 10.2967/jnumed.108.057307 19403881 PMC2755245

[92] Takada K Toyokawa G Yoneshima Y Tanaka K Okamoto I Shimokawa M . 18F-FDG uptake in PET/CT is a potential predictive biomarker of response to anti-PD-1 antibody therapy in non-small cell lung cancer. Sci Rep. (2019) 9:13362. doi: 10.1038/s41598-019-50079-2 31527660 PMC6746703

[93] Bourigault P Skwarski M Macpherson RE Higgins GS McGowan DR . Timing of hypoxia PET/CT imaging after 18F-fluoromisonidazole injection in non-small cell lung cancer patients. Sci Rep. (2022) 12:21746. doi: 10.1038/s41598-022-26199-7 36526815 PMC9758119

[94] Førde D Kilvær T Pedersen MI Blix ES Urbarova I Paulsen EE . High density of TCF1+ stem-like tumor-infiltrating lymphocytes is associated with favorable disease-specific survival in NSCLC. Front Immunol. (2024) 15:1504220. doi: 10.3389/fimmu.2024.1504220 PMC11693705 39749327

[95] Leite da Silva LF Saldanha EF de Menezes JSA Halamy Pereira L de Bragança Dos Santos JAR Buonopane IR . Plasma ctDNA kinetics as a predictor of systemic therapy response for advanced non-small cell lung cancer: A systematic review and meta-analysis. Oncologist. (2025) 30:oyae344. doi: 10.1093/oncolo/oyae344 39998904 PMC11853598

[96] Tonneau M Phan K Manem VSK Low-Kam C Dutil F Kazandjian S . Generalization optimizing machine learning to improve CT scan radiomics and assess immune checkpoint inhibitors' response in non-small cell lung cancer: A multicenter cohort study. Front Oncol. (2023) 13:1196414. doi: 10.3389/fonc.2023.1196414 37546399 PMC10400292

[97] Cousin F Louis T Dheur S Aboubakar F Ghaye B Occhipinti M . Radiomics and delta-radiomics signatures to predict response and survival in patients with non-small-cell lung cancer treated with immune checkpoint inhibitors. Cancers (Basel). (2023) 15:1968. doi: 10.3390/cancers15071968 37046629 PMC10093736

[98] Zhang Z Li Y Yan X Song Q Wang G Hu Y . Pretreatment lactate dehydrogenase may predict outcome of advanced non small-cell lung cancer patients treated with immune checkpoint inhibitors: A meta-analysis. Cancer Med. (2019) 8:1467–73. doi: 10.1002/cam4.2024 30848091 PMC6488146

[99] Honrubia-Peris B Garde-Noguera J García-Sánchez J Piera-Molons N Llombart-Cussac A Fernández-Murga ML . Soluble biomarkers with prognostic and predictive value in advanced non-small cell lung cancer treated with immunotherapy. Cancers (Basel). (2021) 13:4280. doi: 10.3390/cancers13174280 34503087 PMC8428366

[100] Brizel DM Scully SP Harrelson JM Layfield LJ Dodge RK Charles HC . Radiation therapy and hyperthermia improve the oxygenation of human soft tissue sarcomas. Cancer Res. (1996) 56:5347–50. 8968082

[101] Unsoeld M Lamprecht U Traub F Hermes B Scharpf M Potkrajcic V . MR thermometry data correlate with pathological response for soft tissue sarcoma of the lower extremity in a single center analysis of prospectively registered patients. Cancers (Basel). (2020) 12:959. doi: 10.3390/cancers12040959 32295076 PMC7226612

[102] Basch E Reeve BB Mitchell SA Clauser SB Minasian LM Dueck AC . Development of the National Cancer Institute's patient-reported outcomes version of the common terminology criteria for adverse events (PRO-CTCAE). J Natl Cancer Inst. (2014) 106:dju244. doi: 10.1093/jnci/dju244 25265940 PMC4200059

