# Supplementary material for: A lactate–mitochondrial axis framework for mild hyperthermia and checkpoint blockade in driver-negative NSCLC: testing temporal coupling
Source: Front Immunol. 2026 Oct 22;17:1969238. doi: 10.3389/fimmu.2026.1969238 (PMC13736718; doi:10.3389/fimmu.2026.1969238)
Supplement: Supplementary file 1 [file DataSheet1.docx]

# Supplementary Methods: Proposed Operational Specifications for Prospective Testing

This supplement provides the operational details supporting the criteria and phase 0 procedures summarized in Sections 4.3.1, 4.3.3 and 6.1 of the main text. These are specifications proposed for prospective testing, not procedures the authors have carried out. Reference numbers in the Supplementary Methods correspond to those in the main manuscript.

## S1. Operational criteria and evidential levels for a relief window

No consensus threshold currently defines a biologically meaningful relief window. Provisional criteria are therefore proposed at three levels, so that the definition is not validated against the immune outcome it is intended to explain and the strength of each claim matches the directness of the measurement behind it. The first level is the physiological response, the second a candidate metabolic-relief window, and the third mechanistic corroboration and validation; only the third speaks to biological meaningfulness, the first two defining what can be detected above measurement error. Throughout, the relief definition of Section 4.1 is operationalized at the sites where T cells reside: redistribution measured in lymphocyte-dense regions, and not redistribution in general, is what these criteria record. Verification is reserved for quantities measured directly, chiefly intratumoral temperature, and windows themselves are described as detected or recorded.

A physiological window may be considered detected when a prespecified perfusion or oxygenation-sensitive imaging metric changes by more than the repeatability coefficient (RC) of that readout and the change is confirmed at the next scheduled measurement (46, 47). Because the reported persistence of the physiological response ranges from minutes to approximately 1–2 days (8), the measurement schedule and not the biology determines which windows can be observed at all. The schedule proposed below provides an earliest post-session sampling time of approximately 1 h. Under the confirmation rule above, a response first detected at any scheduled time point is recorded as a detected window only if it remains beyond the RC at the next scheduled measurement, so the shortest confirmable duration is set by the interval between the first positive measurement and the next scheduled one rather than by the 1-h sampling time alone. A change exceeding the RC in the prespecified direction at a single post-session time point but absent at the next is recorded as an unconfirmed transient response whose closure time is interval-censored between those two time points, and not as a failure to detect a window; occurrence and duration are separable quantities, and only the second is unresolved here. Isolated RC exceedances may occur by chance when several time points and readouts are examined, so an unconfirmed transient response enters analyses of the core mechanism only where the perfusion and oxygenation-sensitive readouts move together in the prespecified direction at that time point, and is otherwise retained in sensitivity analyses alongside confirmed windows. Its contribution to coverage is bounded by the last measurement at which the response exceeded the RC and the first at which it did not, and is reported as that range in primary mechanistic analyses; any single-value estimate, such as the midpoint, is restricted to sensitivity analysis (48). Denser sampling at the next session is required, now to narrow the censoring interval rather than to rescue an uninterpretable session (Table 2). The term not evaluable is reserved for sessions in which the acquisition failed or did not meet quality criteria, so that four classes of negative observation remain distinct: technical failure of thermal delivery, a session that is not evaluable, an unconfirmed transient response with censored duration, and failure to detect a window despite adequate delivery and sampling.

The RC is estimated from the paired baseline scans of the phase 0 cohort as 2.77 times the pooled within-subject standard deviation, corresponding to the 95% limit of agreement between repeated measurements, because an estimate from two scans in a single patient has very limited precision (47). A pooled within-subject coefficient of variation of 15% for a DCE-MRI transfer-constant measurement yields an RC of approximately 42%, so only a larger change in an individual lesion would be recorded as a detected window (47, 49). Published repeatability estimates for DCE-MRI and for BOLD and TOLD parameters vary substantially with acquisition and analysis method, and adequately described test–retest data remain scarce (50), so each protocol should derive the coefficient from its own baseline scans rather than adopt a literature value, and should state the scale on which it is expressed. The RC defines detectability above measurement error, not a biologically meaningful effect size.

Because the framework requires attenuation of hypoxic constraint and not improved perfusion alone, detected responses are classified further. A response is recorded as hypoxia-consistent where it includes a change in the oxygenation-sensitive readout in the prespecified direction, supported where available by hypoxia-sensitive PET (51), and as perfusion-only where it is confined to the perfusion readout or where the oxygenation-sensitive readout changes but not in that direction. Only hypoxia-consistent responses enter analyses of the core mechanism. Both classes are retained, because oxygenation-sensitive signal is not independent of blood volume and flow, and perfusion-only responses indicate whether perfusion and oxygenation dissociate after heating (50). Both readouts are not required to exceed their RC, since they are correlated and such a rule would raise the false-negative rate without a commensurate gain in specificity.

A candidate metabolic-relief window, more precisely a candidate lactate-associated metabolic-relief window, is recorded when a hypoxia-consistent physiological response co-occurs with a fall, exceeding the RC of that ratio, in the ratio of metabolite-related signal in tumor regions dense in tumor-infiltrating lymphocytes to the tumor-wide signal. The criterion approximates only part of the relief definition adopted here, which specifies redistribution of both lactate and adenosine: the imaging-based ratio addresses lactate-related signal only, and adenosine exposure is assessed indirectly and only where biopsy is available (16), so a candidate window recorded from imaging alone is not evidence that adenosine exposure has fallen. The criterion is deliberately not described as verification, because the measurement is indirect, hyperpolarized ¹³C-pyruvate MRI reporting the conversion of pyruvate rather than extracellular lactate concentration (52), and because a fall in a ratio is arithmetically compatible with an unchanged or rising absolute level in lymphocyte-dense regions. The regional absolute signal and the tumor-wide metabolite burden are therefore reported alongside the ratio as co-primary quantities, and a fall in the ratio accompanied by a rise in the regional absolute signal is not counted as relief. A metabolic-ratio change alongside a perfusion-only response is recorded as a metabolic signal change and does not enter analyses of the core mechanism.

Biopsy-based readouts, such as the spatial density of ectonucleotidase expression or adenosine-pathway signaling in lymphocyte-dense regions relative to remote regions, cannot be repeated at baseline within the same lesion; their reference variability is therefore taken from the spread across multiple fields in the baseline sample, and a change is regarded as supportive when the on-treatment field-level value lies outside the baseline 2.5th to 97.5th percentile range. Because the hypothesis concerns exposure at the sites occupied by T cells, a fall in tumor-wide metabolite burden is not required, provided the regional absolute signal does not rise. The cut-offs and classification rules in this section are provisional and illustrative rather than validated analytical procedures, and the thresholds referred to elsewhere are these. The thresholds referenced in Table 2 of the main text are those specified in this section and are provisional operational criteria rather than validated clinical cut-offs.

The third level is mechanistic corroboration and validation, which is not defined by a threshold and proceeds in two stages that should not be conflated. Corroboration is longitudinal and associational: a candidate metabolic-relief window is regarded as mechanistically supported where changes in metabolic readouts are associated, within patients, with changes in immunological readouts, the anchors being Tpex frequency and T-cell metabolic state. Association does not establish causation, since concurrent changes may equally reflect checkpoint blockade itself, other shifts in the immune microenvironment, or treatment response. Validation therefore depends on three design features, namely randomization of the prespecified schedule with achieved coverage analyzed as a mechanistic exposure, comparison with unheated reference lesions, and ex vivo perturbation of the proposed pathway in sorted tumor-infiltrating T cells. Within this structure, the association of detected physiological windows, of candidate metabolic-relief windows, and of larger changes expressed as multiples of the RC with the immunological readouts is examined as a calibration analysis from which a minimal meaningful effect may be estimated. That analysis, and not the definition of either window, is the evidential basis of any claim that metabolic suppression has been relieved. Measurement is constrained throughout by scale: MRI voxels are considerably coarser than the lymphocyte microenvironment and adenosine exposure cannot at present be imaged directly (53), so co-registration of imaging with multiplexed analysis of paired biopsies is required where biopsy is safe.

Liver reference for the glycolytic measurability gate (Section 6.1). The mean SUL of normal liver is obtained from a 3 cm spherical volume of interest placed in the right hepatic lobe, following PERCIST 1.0 (91). Adapting the PERCIST assessability rule, which was defined for baseline and follow-up comparison, to the paired baseline scans of Section 4.3.3, the two liver reference values are required to agree within 20% and within 0.3 SUL units; a pair that does not meet this requirement is recorded as not evaluable for the glycolytic gate rather than as failing it.

## S2. Sampling schedule for the phase 0 study

Sampling times are prespecified rather than left to convenience, because they determine which windows are detectable at all. Physiological and metabolic readouts would be collected immediately before and at the end of each evaluated session, and afterward at approximately 1, 4, 24, 48 and 72 h. This schedule resolves both the few-hour windows treated as plausible in Section 4.3 and the 1–2-day responses reported in preclinical models (8), and the same times would be used at every evaluated session so that responses are comparable within and between patients. Where the full metabolic acquisition cannot be repeated at every time point, the perfusion and oxygenation-sensitive readouts define the window, and the metabolic acquisition is placed at the expected peak and at one later time, with the resulting asymmetry recorded.

## Supplementary references

8.	Vaupel P, Piazena H, Notter M, Thomsen AR, Grosu AL, Scholkmann F, et al. From Localized Mild Hyperthermia to Improved Tumor Oxygenation: Physiological Mechanisms Critically Involved in Oncologic Thermo-Radio-Immunotherapy. Cancers (Basel). 2023;15(5).

16.	Rodríguez-Pampín I, González-Pico L, Selas A, Andújar A, Prieto-Díaz R, Sotelo E. Targeting the adenosinergic axis in cancer immunotherapy: Insights into A(2A) and A(2B) receptors and novel clinical combination strategies. Pharmacol Rev. 2025;77(6):100092.

46.	Pierce TT, Sirlin CB, Fowler KJ, Buckler AJ, Hall TJ, Obuchowski NA. Understanding Repeatability and Reproducibility Coefficients for Quantitative Imaging Biomarkers. Radiology. 2025;316(2):e250279.

47.	Shukla-Dave A, Obuchowski NA, Chenevert TL, Jambawalikar S, Schwartz LH, Malyarenko D, et al. Quantitative imaging biomarkers alliance (QIBA) recommendations for improved precision of DWI and DCE-MRI derived biomarkers in multicenter oncology trials. J Magn Reson Imaging. 2019;49(7):e101-e21.

48.	Dugué AE, Pulido M, Chabaud S, Belin L, Gal J. How to Deal with Interval-Censored Data Practically while Assessing the Progression-Free Survival: A Step-by-Step Guide Using SAS and R Software. Clin Cancer Res. 2016;22(23):5629-35.

49.	Woodall RT, Sahoo P, Cui Y, Chen BT, Shiroishi MS, Lavini C, et al. Repeatability of tumor perfusion kinetics from dynamic contrast-enhanced MRI in glioblastoma. Neurooncol Adv. 2021;3(1):vdab174.

50.	O'Connor JPB, Robinson SP, Waterton JC. Imaging tumour hypoxia with oxygen-enhanced MRI and BOLD MRI. Br J Radiol. 2019;92(1095):20180642.

51.	Reeves KM, Song PN, Angermeier A, Della Manna D, Li Y, Wang J, et al. (18)F-FMISO PET Imaging Identifies Hypoxia and Immunosuppressive Tumor Microenvironments and Guides Targeted Evofosfamide Therapy in Tumors Refractory to PD-1 and CTLA-4 Inhibition. Clin Cancer Res. 2022;28(2):327-37.

52.	Deen SS, Rooney C, Shinozaki A, McGing J, Grist JT, Tyler DJ, et al. Hyperpolarized Carbon 13 MRI: Clinical Applications and Future Directions in Oncology. Radiol Imaging Cancer. 2023;5(5):e230005.

53.	Jahangir CA, Page DB, Broeckx G, Gonzalez CA, Burke C, Murphy C, et al. Image-based multiplex immune profiling of cancer tissues: translational implications. A report of the International Immuno-oncology Biomarker Working Group on Breast Cancer. J Pathol. 2024;262(3):271-88.

91.	Wahl RL, Jacene H, Kasamon Y, Lodge MA. From RECIST to PERCIST: Evolving Considerations for PET Response Criteria in Solid Tumors. J Nucl Med. 2009;50 Suppl 1:122S-50S.
